# Hierarchical diagnosis of breast phyllodes tumors enabled by deep learning of ultrasound images: a retrospective multi-center study

**DOI:** 10.1186/s40644-025-00879-9

**Published:** 2025-05-08

**Authors:** Yuqi Yan, Yuanzhen Liu, Yao Wang, Tian Jiang, Jiayu Xie, Yahan Zhou, Xin Liu, Meiying Yan, Qiuqing Zheng, Haifei Xu, Jinxiao Chen, Lin Sui, Chen Chen, RongRong Ru, Kai Wang, Anli Zhao, Shiyan Li, Ying Zhu, Yang Zhang, Vicky Yang Wang, Dong Xu

**Affiliations:** 1 Department of Diagnostic Ultrasound Imaging & Interventional Therapy, Zhejiang Cancer Hospital, No.1 East Banshan Road, Gongshu District, Hangzhou, Zhejiang 310022 China; 2 Wenling Institute of Big Data and Artificial Intelligence Institute in Medicine, No.18, Civic Avenue, Wenling, Taizhou, Zhejiang 317502 China; 3 Taizhou Key Laboratory of Minimally Invasive Interventional Therapy & Artificial Intelligence, Taizhou Branch of Zhejiang Cancer Hospital (Taizhou Cancer Hospital), Taizhou, Zhejiang China; 4 Center of Intelligent Diagnosis and Therapy (Taizhou), Hangzhou Institute of Medicine (HIM), Chinese Academy of Sciences, Taizhou, Zhejiang China; 5 Interventional Medicine and Engineering Research Center, Hangzhou Institute of Medicine (HIM), Chinese Academy of Sciences, Hangzhou, Zhejiang China; 6 Postgraduate training base Alliance of Wenzhou Medical University (Zhejiang Cancer Hospital), Hangzhou, Zhejiang China; 7 Department of Ultrasound, Lishui People’s Hospital, Lishui, Zhejiang China; 8 Department of Ultrasound, Zhejiang Xiaoshan Hospital, Hangzhou, Zhejiang China; 9 Department of Ultrasound, The Affiliated Dongyang Hospital of Wenzhou Medical University, Dongyang, Zhejiang China; 10 Department of Ultrasound in Medicine, Affiliated Sir Run Run Shaw Hospital of Zhejiang University School of Medicine, Hangzhou, Zhejiang China; 11 Department of Ultrasound, Ruijin Hospital, Shanghai Jiaotong University School of Medicine, No.197, Ruijin 2nd Road, Huangpu District, Shanghai, Zhejiang 200025 China

**Keywords:** Deep learning, Ultrasound, Breast, Phyllodes tumors, Fibroadenoma

## Abstract

**Objective:**

Phyllodes tumors (PTs) are rare breast tumors with high recurrence rates, current methods relying on post-resection pathology often delay detection and require further surgery. We propose a deep-learning-based Phyllodes Tumors Hierarchical Diagnosis Model (PTs-HDM) for preoperative identification and grading.

**Methods:**

Ultrasound images from five hospitals were retrospectively collected, with all patients having undergone surgical pathological confirmation of either PTs or fibroadenomas (FAs). PTs-HDM follows a two-stage classification: first distinguishing PTs from FAs, then grading PTs into benign or borderline/malignant. Model performance metrics including AUC and accuracy were quantitatively evaluated. A comparative analysis was conducted between the algorithm’s diagnostic capabilities and those of radiologists with varying clinical experience within an external validation cohort. Through the provision of PTs-HDM’s automated classification outputs and associated thermal activation mapping guidance, we systematically assessed the enhancement in radiologists’ diagnostic concordance and classification accuracy.

**Results:**

A total of 712 patients were included. On the external test set, PTs-HDM achieved an AUC of 0.883, accuracy of 87.3% for PT vs. FA classification. Subgroup analysis showed high accuracy for tumors < 2 cm (90.9%). In hierarchical classification, the model obtained an AUC of 0.856 and accuracy of 80.9%. Radiologists’ performance improved with PTs-HDM assistance, with binary classification accuracy increasing from 82.7%, 67.7%, and 64.2–87.6%, 76.6%, and 82.1% for senior, attending, and resident radiologists, respectively. Their hierarchical classification AUCs improved from 0.566 to 0.827 to 0.725–0.837. PTs-HDM also enhanced inter-radiologist consistency, increasing Kappa values from − 0.05 to 0.41 to 0.12 to 0.65, and the intraclass correlation coefficient from 0.19 to 0.45.

**Conclusion:**

PTs-HDM shows strong diagnostic performance, especially for small lesions, and improves radiologists’ accuracy across all experience levels, bridging diagnostic gaps and providing reliable support for PTs’ hierarchical diagnosis.

**Supplementary Information:**

The online version contains supplementary material available at 10.1186/s40644-025-00879-9.

## Key points

• Accurate preoperative diagnosis and grading of Phyllodes tumors remain challenging.

• An ultrasound-based deep learning model enables preoperative hierarchical diagnosis of Phyllodes tumors.

• PTs-HDM improves radiologists’ accuracy and consistency, reducing diagnostic variability.

**Supplementary Information:**

The online version contains supplementary material available at 10.1186/s40644-025-00879-9.

## Introduction

Phyllodes tumors (PTs) are rare fibroepithelial breast neoplasms, accounting for 2.5% of fibroepithelial lesions, with fibroadenomas (FAs) comprising the remainder [1]. Based on histological features including stromal cellularity, atypia, and mitotic activity, the WHO classifies PTs into benign, borderline, and malignant subtypes, which exhibit progressively increasing recurrence rates of 7.1%, 16.7%, and 25.1%, respectively [2, 3]. This classification directly guides treatment approaches: benign PTs require simple excision, while borderline and malignant variants necessitate wide excision margins and potential adjuvant radiotherapy. In contrast, FAs typically only require monitoring after diagnosis, underscoring the critical importance of accurate preoperative differentiation and grading [4].

Currently, preoperative PT diagnosis relies on pathological and imaging examinations, both of which face accuracy challenges. Pathologically, Fine needle aspiration has limited utility due to overlapping features between PTs and FAs [5]. Although core needle biopsy (CNB) provides more detailed information, tumor heterogeneity often leads to misclassification, with positive predictive values (PPV) ranging from 65 to 83% for PTs [6, 7]. From an imaging perspective, ultrasound is widely used due to being radiation-free and cost-effective. While lobulated appearance and heterogeneous echoes are more common in PTs [8], these features are not consistently observed, and diagnostic accuracy heavily depends on radiologists’ expertise, with typical accuracy rates below 71% [9]. These challenges underscore the urgent need for innovative and reliable tools to enhance preoperative diagnosis and grading of PTs.

In response to these challenges, deep learning (DL) has emerged as a promising solution, demonstrating superior capability in extracting complex imaging features and identifying subtle patterns that may elude human observation [10–15]. Despite these advancements, the application of DL models to ultrasound imaging for differentiating PTs from FAs remains underexplored. To date, few studies have investigated this approach, and none have addressed the use of DL for grading PTs [9, 16, 17]. This gap highlights the need for further research to harness DL’s potential in improving the preoperative differentiation and grading of PTs, paving the way for more precise and personalized treatment strategies.

To this end, this study developed and validated a Phyllodes Tumors Hierarchical Diagnosis Model (PTs-HDM) driven by DL techniques and assessed its applicability using a multicenter ultrasound dataset. PTs-HDM consists of two stages: distinguishing PTs from FAs and grading PTs into borderline/malignant or benign categories. To assess its clinical utility in terms of enhancing diagnostic accuracy and reducing classification inconsistencies, we recruited six radiologists with varying expertise in ultrasound to evaluate their diagnostic performance in two rounds of reader studies, both with and without PTs-HDM assistance.

## Materials & methods

This multi-center diagnostic study adhered to the principles outlined in the Declaration of Helsinki and received approval from the ethics committee of all participating institutions (details in Appendix S1). Given the retrospective nature of this study, the requirement for individual consent was waived.

### Participants and datasets

**Fig. 1:**
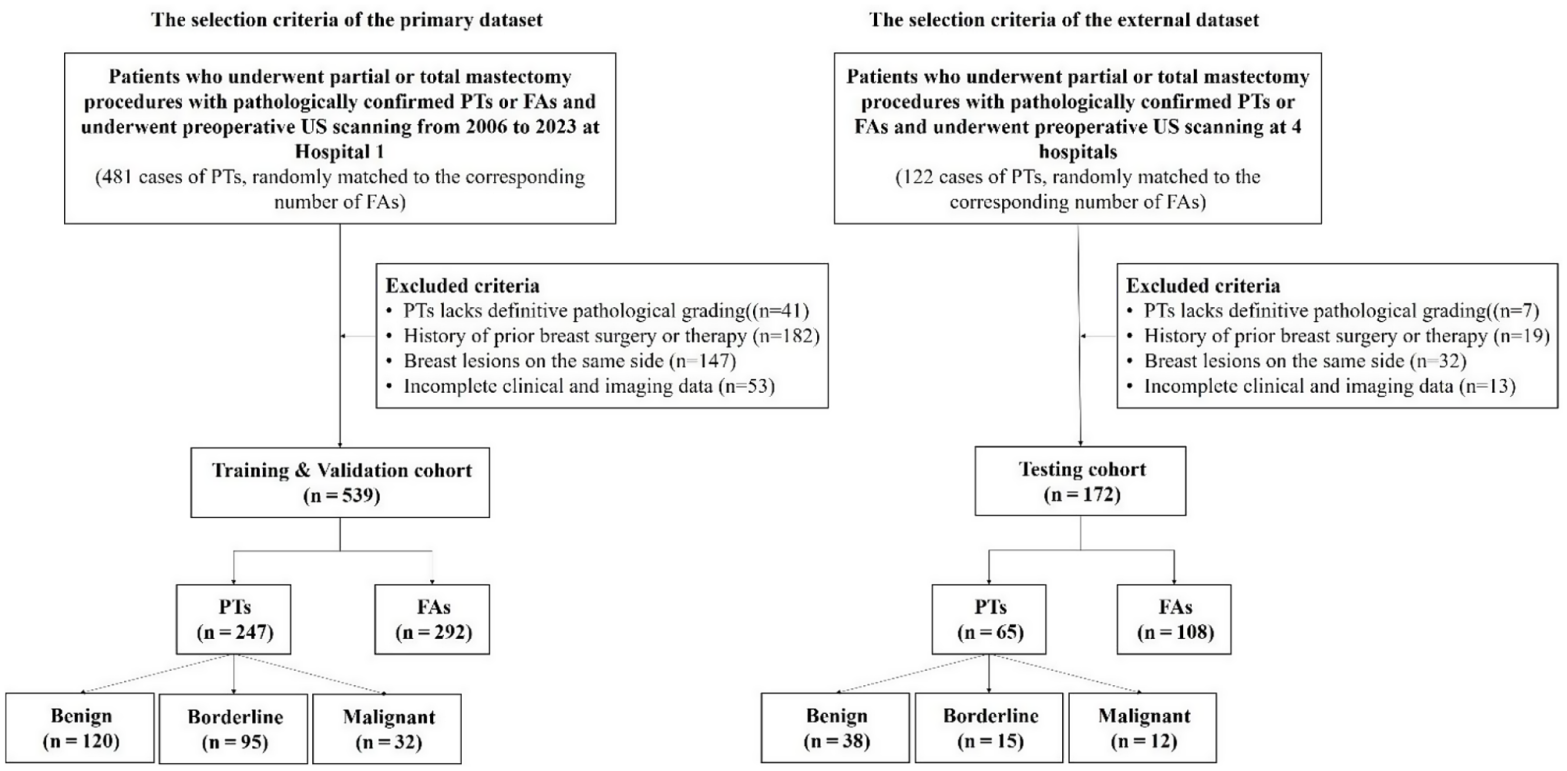
Patient selection flowchart. Hospital 1, Zhejiang Cancer Hospital; FAs, fibroadenomas; PTs, phyllodes tumors; US, ultrasound

This study involved patients from five tertiary referral hospitals located in eastern China: Zhejiang Cancer Hospital (Hospital 1), and four additional hospitals (Hospitals 2–5). A total of 519 patients from Hospital 1, treated between January 2006 and May 2023, were included in the training and internal validation cohort, while 173 patients from Hospitals 2–5, treated between 2021 and 2024, made up the external test cohort. Patients were retrospectively and consecutively screened through the pathological database. To prevent excessive data bias from affecting the model, we randomly matched an equal number of fibroadenomas to phyllodes tumors in the database during inclusion. Additionally, for each patient’s available imaging data, we retained all accessible ultrasound images to facilitate the learning of comprehensive tumor-related features. The patient enrollment process is summarized in Fig. 1. The inclusion and exclusion criteria are as follows.

## Inclusion criteria included

- Patients diagnosed with either PTs or FAs, with histological confirmation and PT grading performed post-surgery by pathologists with over 10 years of experience.
- Patients who underwent US examination within two weeks prior to surgical excision.

## Exclusion criteria included

- PT cases lacking definitive pathological grading.
- prior history of breast surgery or therapy.
- Multiple breast lesions on the same side.
- Incomplete clinical or imaging data.

A total of 12 different ultrasound diagnostic instruments were used to image the patients included in this study (see Fig. S1). Baseline characteristics (i.e. age, gender, menopausal status) of patients and lesions (such as location and size) were obtained from the electronic medical record system.

## Study design

**Fig. 2:**
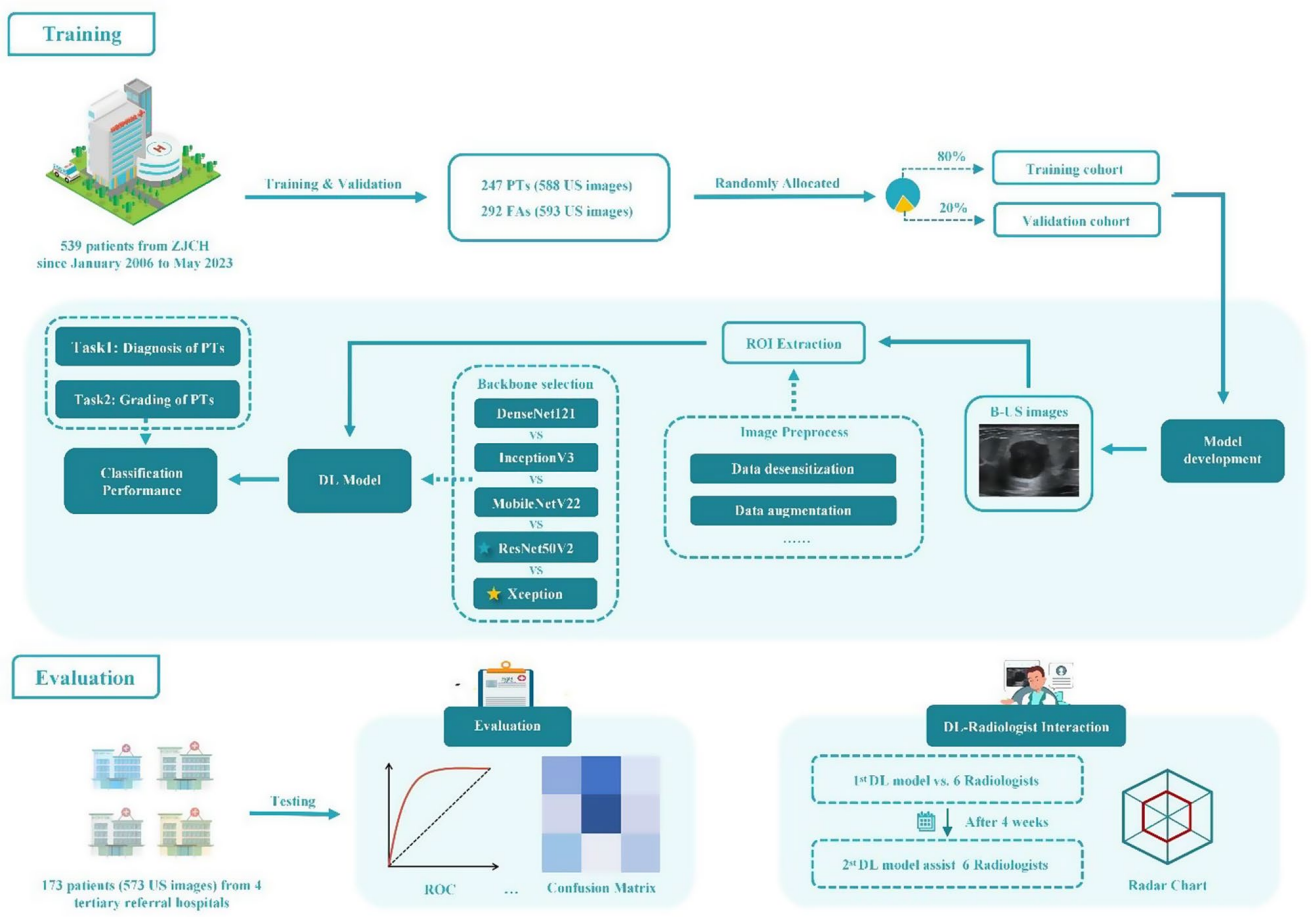
Overview of the study. Phase 1 (Model training): 1181 ultrasound images were collected from one single hospital, and five deep learning network models with different structures were trained and validated to construct a phyllodes tumors hierarchical diagnosis model (PTs-HDM), which iteratively performed the tasks of PTs diagnosis (i.e. to discriminate between FAs and PTs), and PTs grading (to distinguish benign PTs from borderline/malignant PTs). Phase 2 (Evaluation): Data from four other hospitals were collected as the external test set for evaluating the performance of the models. Phase 3 (DL-Radiologist interaction): Finally, the diagnostic performance of six radiologists with and without input from DL models was evaluated. Yellow stars indicate the optimal backbone model for PTs diagnosis; green stars indicate the optimal backbone model for PTs grading. FAs, fibroadenomas; PTs, phyllodes tumors; B-US, Breast Ultrasound; DL, deep learning; ROI, region of interest; ROC, receiver operating characteristic

This multicenter, retrospective diagnostic study comprised three sequential phases. In Phase 1 (Fig. 2: Training), a DL model was developed using grayscale ultrasound images with a two-stage diagnostic framework: the first stage differentiated PTs from FAs, while the second stage classified PTs into benign or borderline/malignant categories. Phase 2 (Fig. 2: Evaluation), validated the model’s diagnostic performance using an external test dataset. In Phase 3 (Fig. 2: DL-Radiologist Interaction), the model’s accuracy was compared with that of radiologists using the same external test dataset to assess its potential as a decision-support tool.

## Data preprocessing

All grayscale ultrasound images in DICOM format were de-identified using custom Python scripts to ensure patient privacy. For each patient, a radiologist with 6 years of ultrasound experience selected representative images (1–4 images per tumor) and annotated the lesion areas using Labelme annotation software (https://github.com/labelmeai/labelme). These labels were then used in Python-based cropping scripts to isolate the lesion regions from the original images.

To augment the training dataset, various data augmentation techniques were implemented, including vertical/horizontal flipping, image rotation (5°-15° range), and random cropping. This approach resulted in a fourfold expansion of the dataset (from 1,181 to 4,724 images), thereby enhancing the model’s robustness through increased training data diversity.

### Model development

**Fig. 3:**
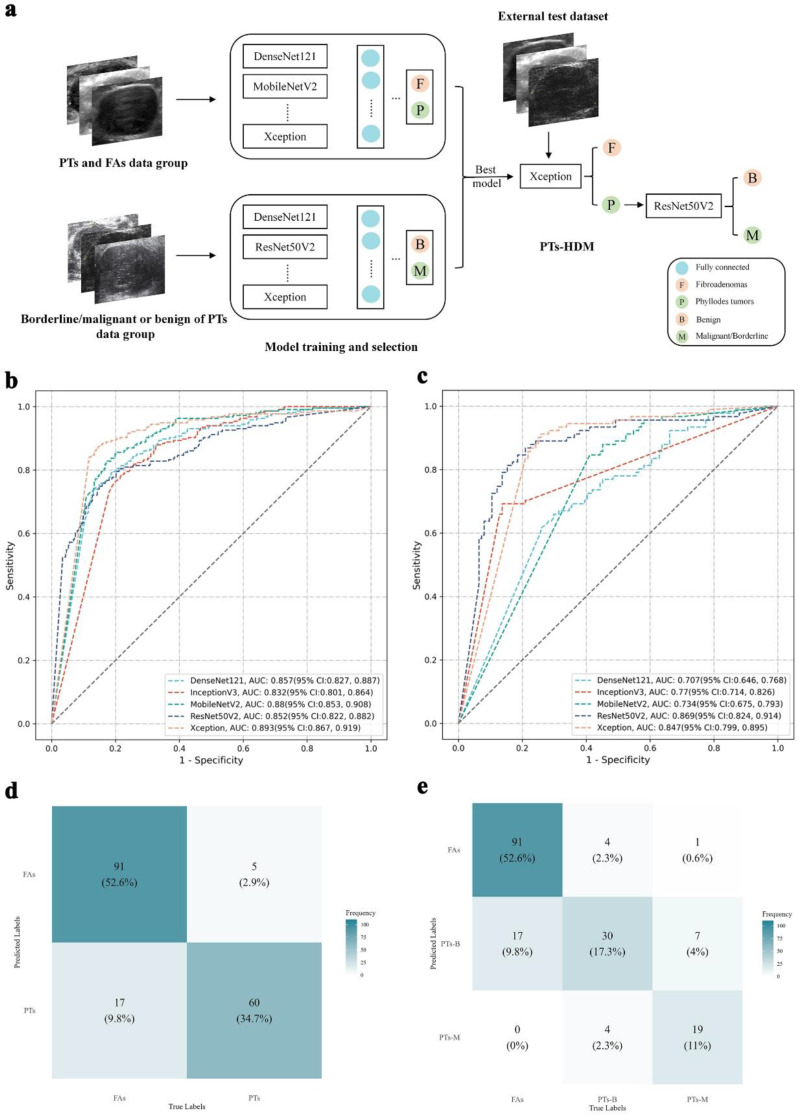
Structure and diagnostic performance of PTs-HDM. **a**) Overall architecture diagram of PTs-HDM; **b**) ROC curve for the fibroadenomas vs. phyllodes tumor classification model; **c**) ROC curve for the borderline/malignant vs. benign classification model; **d**) Confusion matrix for the binary classification of phyllodes tumors vs. fibroadenomas using PTs-HDM; **e**) Confusion matrix for the hierarchical diagnosis of borderline/malignant PTs, benign PTs, and FAs using PTs-HDM. FAs, fibroadenomas; PTs, phyllodes tumors;-B, Benign; -M, Borderline/Malignant; PTs-HDM, phyllodes tumors hierarchical diagnosis model

PTs-HDM is a two-stage classification model. The first stage (diagnostic network) differentiates PTs from FAs, and the second stage (grading network) classifies PTs as borderline/malignant or benign. The overall structure of PTs-HDM is shown in Fig. 3a. After evaluating multiple convolutional neural networks (DenseNet121, InceptionV3, MobileNetV2, ResNet50V2, and Xception), we selected Xception for diagnosis and ResNet50V2 for grading based on performance metrics (Fig. 3b and c, Table S1, S2). Detailed training protocols are available in Appendix S2. In the first stage, lesions classified as FAs were excluded from further analysis. Lesions identified as PTs proceeded to stage two for grading (borderline/malignant vs. benign). The model’s overall performance was evaluated by combining the metrics from both stages. PTs-HDM operates solely on ultrasound images without additional patient data.

To enhance the interpretability of the model, Gradient-weighted Class Activation Mapping (Grad-CAM) was applied to visualize the final classification layer. Grad-CAM highlighted regions of interest in the ultrasound images that contributed to the model’s predictions, with color coding from red (highest attention) to blue (lowest attention), providing insight into the model’s decision-making process.

### DL-Radiologist interaction

A two-round radiologist study was conducted to assess the diagnostic performance of PTs-HDM and its clinical value. Six radiologists with varying ultrasound experience (3–11 years, average 7 years) participated in a two-round diagnostic study. They were categorized as senior radiologists (WY, XHF), attending radiologists (YMY, CXH), and residents (YYQ, TJ), with WY and YYQ specializing in breast ultrasound and others in general ultrasound. All radiologists independently evaluated shuffled cases from the external test cohort through an online platform (Wenjuanxing), blinded to pathological information.

Round one involved diagnosis based solely on ultrasound images and patient baseline data (age, location, size), with radiologists classifying tumors as fibroadenomas, benign PTs, or borderline/malignant PTs. After a four-week interval, round two provided additional PTs-HDM predictions, including probability scores and heatmaps. Radiologists could maintain or revise their initial diagnoses. The study assessed improvements in diagnostic accuracy and consistency with PTs-HDM assistance.

### Statistical analysis

All DL models were trained using TensorFlow-GPU (version 2.6.0), and statistical analyses were performed in Python (version 3.8.15). Non-normally distributed continuous data were summarized as M (Q₁, Q₃) and compared using rank sum test. Categorical variables were compared using chi-square or Fisher’s exact test.

Model performance was evaluated using Receiver Operating Characteristic (ROC) curves, Decision Curve Analysis (DCA) and confusion matrices on the external test set. Confusion matrices were constructed to compare diagnostic outcomes between PTs-HDM and radiologists. A two-sided DeLong test was employed to assess statistical differences in the area under the ROC curve (AUC) between groups. Diagnostic performance metrics, including accuracy, sensitivity, specificity, PPV, negative predictive value (NPV), and F1-score, were calculated using the Scikit-learn library (version 1.1.3). For multi-class classification tasks, additional metrics such as recall, precision, and F1-score were applied. Bootstrap method provided 95% CIs for performance metrics. Inter-reader agreement was assessed using intraclass correlation coefficient (ICC) and Cohen’s kappa coefficients, with significance tested using asymptotic standard errors (null hypothesis κ = 0). Statistical significance was set at *P* < 0.05.

## Results

### Baseline characteristics

This multicenter study included 712 patients (Fig. 1). The training cohort from Hospital 1 comprised 1181 images of 519 patients (292 FAs, median age 38.0 years; 247 PTs, median age 46.0 years). The external test cohort from four hospitals included 537 images of 133 patients (108 FAs, median age 39.0 years; 65 PTs, median age 46.0 years). Age, lesion diameter, and menopausal status showed no significant differences between groups (*P* > 0.05). Detailed patient demographics and lesion characteristics are summarized in Table 1, while the number, demographics, and lesion details for each pathology type are provided in Table S3.

**Table 1:** Clinical and imaging characteristics of the training & validation set, and the external test set

Characteristics	Total	Training & Validation	External Test	*P*
Patients (n)	712	539	173	-
Images (n)	1718	1181	537	-
Age (y), M (Q₁, Q₃)	42.0 (33.0, 50.0)	41.0 (33.0, 51.0)	42.0 (33.0, 50.0)	0.59
Menstrual status (n, %)				0.42
Postmenopausal	157 (22.1)	42 (24.3)	115 (21.3)	
Premenopausal	555 (77.9)	131 (75.7)	424 (78.7)	
Lesion diameter (mm), M (Q₁, Q₃)	23.0 (16.0, 34.0)	21.0 (15.0, 32.0)	24.0 (17.0, 34.0)	0.42

P-value is the result of the comparison between the Training & Validation and Testing groups. PTs, phyllodes tumors; FAs, fibroadenomas; M: Median, Q₁: 1st Quartile, Q₃: 3st Quartile

### PTs-HDM performance evaluation

The Xception and ResNet50V2 networks were selected as the backbone architectures for PTs-HDM. The external test cohort was used to independently evaluate the performance of the DL models. The stage-one model (Xception), designed to differentiate between PTs and FAs, achieved AUC of 0.893 (95% CI: 0.867–0.919), with an accuracy of 86.1% (95% CI: 81.0% − 91.3%), sensitivity of 83.3% (95% CI: 77.8% − 88.9%), and specificity of 90.8%. The stage-two model (ResNet50V2), responsible for distinguishing borderline/malignant PTs from benign PTs, reported an AUC of 0.869 (95%CI: 0.824–0.914), with an accuracy, sensitivity, and specificity of 80.0% (95%CI: 70.3% − 89.7%), 86.8% (95%CI: 78.6% − 95.1%), and 70.4% (95%CI: 59.3% − 81.5%), respectively. Corresponding ROC curves are presented in Fig. 3b and c. Detailed performance metrics are provided in Table S1. When evaluated on the external test set, PTs-HDM achieved a micro-AUC of 0.856 (95% CI: 0.809–0.900), and accuracy of 80.9% (95% CI: 74.6% − 86.7%). The confusion matrix for the external test set is shown in Fig. 3d and e. The performance metrics for the micro and macro methods are shown in Tables 3, and the metrics for the weighted method are shown in Table S4. As evidenced by DCA, the PTs-HDM demonstrated clinical utility in differentiating PTs from FAs when the probability threshold was set between 10% and 75%, where its decision curve consistently remained above both the ‘none’ and ‘all’ intervention reference lines (Fig. S2a). Similarly, for distinguishing borderline/malignant PTs from benign PTs, the model maintained diagnostic validity across a threshold range of 15-75% (Fig. S2b).

### Heatmap-Based model interpretability

Heatmap visualization was employed to elucidate the decision-making process of PTs-HDM. Performance differences were observed between cases correctly and incorrectly predicted (Fig. S3). In correctly classified cases, the model focused on critical internal lesion features across all pathological types. Misclassified cases showed inadequate capture of key diagnostic features. Radiologists were advised that model predictions might be unreliable when heatmaps focused beyond tumor edges or showed predominantly blue regions, warranting clinical judgment.

### Diagnostic performance of radiologists

**Fig. 4:**
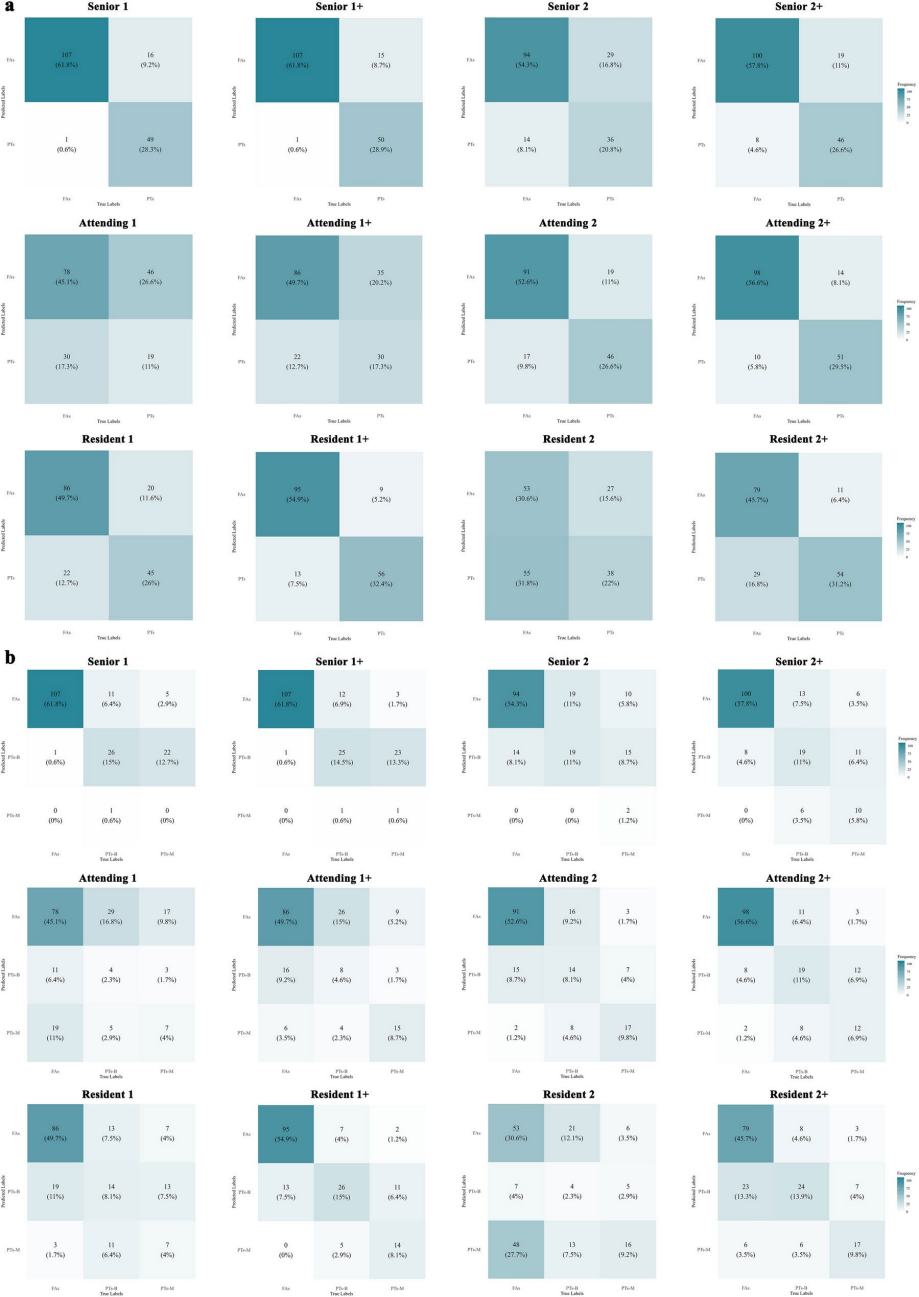
Confusion matrices of six radiologists performing hierarchical diagnosis and binary classification with and without PTs-HDM assistance. **a**) Binary Classification: Each matrix compares the performance of the same six radiologists for differentiating between fibroadenomas and phyllodes tumors. **b**) Hierarchical Diagnosis: Each matrix represents the distribution of predictions for FAs, benign PTs, and borderline/malignant PTs across six radiologists. Rows indicate the actual labels, and columns indicate the predicted labels. Across both binary classification and hierarchical diagnosis tasks, PTs-HDM assistance improved diagnostic accuracy, reducing misclassification rates and increasing consistency, especially for borderline/malignant cases (PTs-M) This effect was more pronounced for residents compared to seniors and attendings, reflecting the potential of PTs-HDM to augment less experienced radiologists. FAs, fibroadenomas; PTs, phyllodes tumors; -B, Benign; -M, Borderline/Malignant; +, with PTs-HDM assistance

In binary classification (PTs vs. FAs), senior radiologists achieved accuracy of 82.7% (95% CI: 77.5-87.9%), sensitivity of 76.9% (95% CI: 65.7-87.0%), and specificity of 99.1% (95% CI: 96.8-100.0%), outperforming attending radiologists and residents (Table 2). Detailed performance is shown in Fig. 4a.

**Table 2:** Comparison of diagnostic performance for PTs and FAs among 6 radiologists, and between radiologists with and without PTs-HDM assistance

	AUC	Accuracy	Sensitivity	Specificity	PPV	NPV	F1-score
PTs-HDM	0.883 (0.831, 0.927)	87.3 (82.1, 91.9)	92.3 (84.9, 98.4)	84.3 (76.6, 90.4)	77.9 (68.1, 86.7)	94.8 (89.8, 98.9)	84.5 (77.5, 90.3)
Senior 1	0.872 (0.821, 0.925)	90.2 (86.1, 94.2)	75.4 (65.1, 85.7)	99.1 (97.1, 100.0)	98.0 (93.3, 100.0)	87.0 (81.0, 92.8)	85.2 (78.1, 91.7)
Senior 1+	0.880 (0.826, 0.931) ↑	90.8 (86.7, 94.8) ↑	76.9 (65.7, 87.0) ↑	99.1 (96.8, 100.0)	98.0 (93.2, 100.0)	87.7 (81.7, 93.2) ↑	86.2 (78.4, 92.6) ↑
Senior 2	0.712 (0.647, 0.780)	75.1 (68.8, 81.5)	55.4 (43.8, 67.5)	87.0 (80.4, 93.2)	72.0 (59.6, 84.1)	76.4 (69.2, 83.7)	62.6 (51.9, 72.3)
Senior 2 + AI	0.817 (0.755, 0.880) ↑	84.4 (79.2, 89.6) ↑	70.8 (59.7, 82.4) ↑	92.6 (87.0, 97.2) ↑	85.2 (74.6, 94.2) ↑	84.0 (77.8, 90.3) ↑	77.3 (68.4, 85.7) ↑
Senior Mean	0.792 (0.734, 0.853)	82.7 (77.5, 87.9)	65.4 (54.5, 76.6)	93.1 (88.8, 96.6)	85.0 (76.5, 92.1)	81.7 (75.1, 88.3)	73.9 (65.0, 82.0)
Senior Mean+	0.848 (0.789, 0.906) ↑	87.6 (83.0, 92.2) ↑	73.9 (62.7, 84.7) ↑	95.8 (91.9, 98.6) ↑	91.6 (83.9, 97.1) ↑	85.9 (79.8, 91.8) ↑	81.8 (73.4, 89.2) ↑
Attending 1	0.507 (0.441, 0.585)	56.1 (49.1, 64.2)	29.2 (18.9, 41.0)	72.2 (63.5, 81.0)	38.8 (25.0, 54.2)	62.9 (54.6, 71.7)	33.3 (22.0, 45.0)
Attending 1 + AI	0.629 (0.556, 0.695) ↑	67.1 (60.1, 73.4) ↑	46.2 (33.3, 58.5) ↑	79.6 (71.7, 86.5) ↑	57.7 (44.4, 70.2) ↑	71.1 (62.7, 78.8) ↑	51.3 (39.2, 61.0) ↑
Attending 2	0.775 (0.713, 0.839)	79.2 (73.4, 85.0)	70.8 (59.5, 81.7)	84.3 (76.8, 90.8)	73.0 (61.0, 84.1)	82.7 (75.7, 89.3)	71.9 (62.6, 80.0)
Attending 2 + AI	0.846 (0.787, 0.904) ↑	86.1 (80.9, 91.3) ↑	78.5 (68.1, 87.9) ↑	90.7 (84.9, 96.1) ↑	83.6 (74.6, 92.6) ↑	87.5 (81.7, 93.2) ↑	81.0 (72.9, 88.1) ↑
Attending Mean	0.641 (0.577, 0.712)	67.7 (61.3, 74.6)	50.0 (39.2, 61.4)	78.3 (70.2, 85.9)	55.9 (43.0, 69.2)	72.8 (65.2, 80.5)	52.6 (42.3, 62.5)
Attending Mean+	0.738 (0.672, 0.800) ↑	76.6 (70.5, 82.4) ↑	62.4 (50.7, 73.2) ↑	85.2 (78.3, 91.3) ↑	70.7 (59.5, 81.4) ↑	79.3 (72.2, 86.0) ↑	66.2 (56.1, 74.6) ↑
Resident 1	0.744 (0.675, 0.813)	75.7 (69.4, 82.1)	69.2 (58.3, 81.0)	79.6 (71.7, 86.9)	67.2 (54.8, 78.8)	81.1 (73.5, 88.6)	68.2 (58.6, 77.1)
Resident 1 + AI	0.871 (0.817, 0.921) ↑	87.3 (82.1, 91.9) ↑	86.2 (78.0, 93.8) ↑	88.0 (81.5, 93.5) ↑	81.2 (71.1, 90.0) ↑	91.3 (86.0, 96.3) ↑	83.6 (76.3, 89.7) ↑
Resident 2	0.538 (0.478, 0.614)	52.6 (44.7, 60.7)	58.5 (45.7, 70.5)	49.1 (40.0, 59.4)	40.9 (31.4, 50.5)	66.3 (55.6, 76.9)	48.1 (38.6, 57.3)
Resident 2 + AI	0.781 (0.718, 0.840) ↑	76.9 (70.5, 82.7) ↑	83.1 (73.3, 92.1) ↑	73.1 (64.4, 81.1) ↑	65.1 (54.8, 74.7) ↑	87.8 (80.4, 94.1) ↑	73.0 (64.4, 81.5) ↑
Resident Mean	0.641 (0.577, 0.714)	64.2 (57.1, 71.4)	63.9 (52.0, 75.8)	64.4 (55.9, 73.2)	54.1 (43.1, 64.7)	73.7 (64.6, 82.8)	58.2 (48.6, 67.2)
Resident Mean+	0.826 (0.768, 0.881) ↑	82.1 (76.3, 87.3) ↑	84.7 (75.7, 93.0) ↑	80.6 (73.0, 87.3) ↑	73.2 (63.0, 82.4) ↑	89.6 (83.2, 95.2) ↑	78.3 (70.4, 85.6) ↑

The data in brackets represent the 95% confidence intervals PPV, positive predictive value; NPV, negative predictive value; + indicates with PTs-HDM assistance The upward arrow (↑) represents indicators that improved owing to PTs-HDM assistance

In the hierarchical diagnosis of PTs (FAs, benign PTs, malignant PTs), senior radiologists demonstrated significantly superior diagnostic performance compared to attending radiologists, who, in turn, outperformed residents (micro-AUC: 0.788, [95% CI: 0.736–0.838] vs. micro-AUC: 0.708, [95% CI: 0.656–0.762] vs. micro-AUC: 0.641, [95% CI: 0.588–0.695]). The breast ultrasound specialist represented by senior radiologist 1 and resident 1 exhibited markedly better performance compared to their counterparts of equivalent seniority without breast ultrasound specialization (Senior micro-AUC: 0.827 vs. 0.749, Resident micro-AUC: 0.715 vs. 0.566). Figure 4b presents the confusion matrices, showing the quantity and percentage of correct predictions in the three-class classification. Furthermore, PTs-HDM achieved micro-AUC of 0.856 (0.809-0.900), surpassing attending radiologists and residents, comparable to senior breast specialists (Table 3). Weighted hierarchical diagnostic indicators are presented in Table S4.

**Table 3:** Comparison of diagnostic performance between PTs-HDM and 6 radiologists

	AUC-micro	AUC-macro	Accuracy-macro	Recall-macro	Precision-macro	F1-macro
PTs-HDM	0.856 (0.809, 0.900)	0.842 (0.787, 0.893)	80.9 (75.1, 86.7)	77.8 (69.8, 85.2)	78.0 (70.9, 84.2)	76.6 (69.1, 83.6)
Senior 1	0.827 (0.779, 0.874)	0.709 (0.668, 0.746)	76.8 (70.5, 83.2)	55.8 (50.7, 60.9)	46.7 (41.7, 51.3)	50.7 (45.9, 54.9)
Senior 1+	0.827 (0.779, 0.874)	0.680 (0.637, 0.723)	76.8 (70.5, 83.2)	56.2 (50.8, 62.0) ↑	61.4 (42.3, 82.7) ↑	52.1 (46.0, 59.2) ↑
Senior 2	0.749 (0.692, 0.801)	0.631 (0.578, 0.681)	66.5 (59.0, 72.8)	48.2 (41.5, 54.5)	67.5 (36.0, 76.8)	46.2 (39.1, 54.8)
Senior 2+	0.810 (0.757, 0.857) ↑	0.720 (0.663, 0.777) ↑	74.5 (67.6, 80.3) ↑	59.7 (51.7, 68.4) ↑	65.3 (55.3, 74.9)	61.3 (52.2, 70.2) ↑
Senior Mean	0.788 (0.736, 0.838)	0.670 (0.623, 0.714)	71.7 (64.8, 78.0)	52.0 (46.1, 57.7)	57.1 (38.9, 64.1)	48.5 (42.5, 54.9)
Senior Mean+	0.819 (0.768, 0.866) ↑	0.700 (0.650, 0.750) ↑	74.3 (68.8, 80.2) ↑	56.0 (49.2, 62.8) ↑	54.5 (47.1, 62.0)	54.4 (47.3, 61.3) ↑
Attending 1	0.636 (0.584, 0.692)	0.518 (0.468, 0.573)	51.4 (43.4, 58.4)	36.4 (29.7, 43.1)	36.0 (28.0, 45.0)	34.8 (27.5, 42.6)
Attending 1+	0.725 (0.671, 0.775) ↑	0.635 (0.575, 0.694) ↑	63.1 (55.5, 70.0) ↑	52.0 (44.1, 59.7) ↑	53.2 (44.2, 62.3) ↑	52.3 (43.5, 60.3) ↑
Attending 2	0.780 (0.727, 0.831)	0.720 (0.657, 0.781)	70.5 (63.6, 77.5)	61.3 (52.5, 69.8)	61.6 (52.8, 70.2)	61.2 (53.1, 68.8)
Attending 2+	0.809 (0.762, 0.853) ↑	0.737 (0.679, 0.793) ↑	74.6 (67.6, 80.9) ↑	61.9 (53.0, 70.5) ↑	63.3 (54.3, 71.9) ↑	62.3 (53.7, 71.2) ↑
Attending Mean	0.708 (0.656, 0.762)	0.619 (0.566, 0.675)	61.0 (53.5, 68.0)	48.9 (41.1, 56.5)	48.8 (40.4, 57.6)	48.0 (40.3, 55.7)
Attending Mean+	0.767 (0.717, 0.814) ↑	0.686 (0.627, 0.744) ↑	68.9 (61.6, 80.9) ↑	61.9 (53.0, 70.5) ↑	63.3 (54.3, 71.9) ↑	62.3 (53.7, 71.2) ↑
Resident 1	0.715 (0.662, 0.766)	0.630 (0.575, 0.690)	61.8 (54.3, 69.4)	47.5 (39.5, 55.6)	48.2 (40.3, 56.5)	47.3 (39.6, 54.8)
Resident 1+	0.837 (0.792, 0.883) ↑	0.789 (0.732, 0.842) ↑	78.1 (72.3, 83.8) ↑	69.5 (61.2, 78.2) ↑	72.5 (63.6, 80.6) ↑	69.8 (61.3, 77.8) ↑
Resident 2	0.566 (0.514, 0.623)	0.544 (0.483, 0.601)	42.4 (35.3, 49.1)	39.9 (31.9, 47.2)	37.3 (29.4, 46.1)	34.0 (27.4, 41.2)
Resident 2+	0.770 (0.714, 0.818) ↑	0.753 (0.694, 0.810) ↑	69.5 (63.0, 76.3) ↑	66.3 (57.9, 74.6) ↑	63.7 (55.3, 71.5) ↑	64.0 (55.6, 72.0) ↑
Resident Mean	0.641 (0.588, 0.695)	0.587 (0.529, 0.646)	52.1 (44.8, 59.3)	43.7 (35.7, 51.4)	42.8 (34.9, 51.3)	40.7 (33.5, 48.0)
Resident Mean+	0.804 (0.753, 0.851) ↑	0.771 (0.713, 0.826) ↑	73.8 (67.7, 80.1) ↑	67.9 (59.6, 76.4) ↑	68.1 (59.5, 76.1) ↑	66.9 (58.5, 74.9) ↑

The data in brackets represent the 95% confidence intervals. + indicates with PTs-HDM assistance. The upward arrow (↑) represents indicators that improved owing to AI assistance

### Diagnostic performance improvement with PTs-HDM assistance

In the binary classification of PTs vs. FAs, radiologists’ AUC values (0.507–0.872) improved with PTs-HDM assistance (0.629–0.880). Mean accuracy increased from 82.7 to 87.6% for senior radiologists, 67.7–76.6% for attending radiologists, and 64.2–82.1% for residents (Table 2 and Fig. S4). Comparable trends were noted in the hierarchical diagnosis of PTs, micro-AUC values improved from 0.566 to 0.827 to 0.725–0.837 with PTs-HDM assistance. Notably, with PTs-HDM support, residents and attending radiologists achieved diagnostic performance comparable to senior radiologists, with Resident 2 demonstrating a substantial AUC improvement of 0.204 underscores the model’s potential to reduce the impact of experience disparity. Similar improvements were observed across all radiologists in terms of accuracy, recall, precision, and F1-score (Table 3). In conclusion, PTs-HDM enhanced diagnostic performance across all radiologist levels, effectively bridging experience gaps.

### Enhanced diagnostic consistency with PTs-HDM integration

**Fig. 5:**
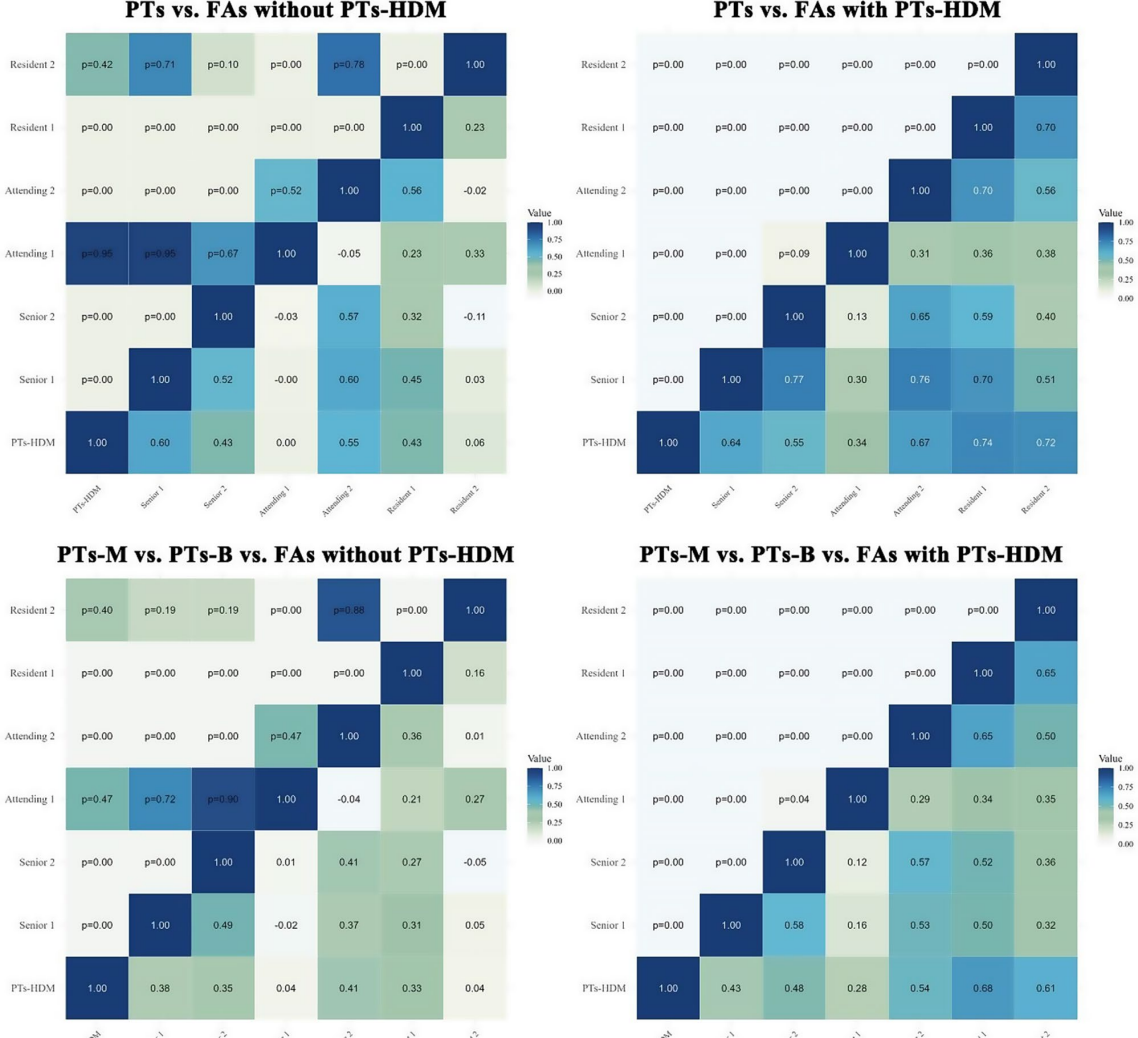
Heatmaps showing the inter-rater agreement among different participants across four scenarios. (1) PTs vs. FAs without PTs-HDM (top-left); (2) PTs vs. FAs with PTs-HDM (top-right), (3) PTs-M vs. PTs-B vs. FAs without PTs-HDM (bottom-left), (4) PTs-M vs. PTs-B vs. FAs with PTs-HDM (bottom-right). Kappa values were interpreted according to Landis and Koch’s guidelines: ≤0 indicates no agreement, 0.01–0.20 slight agreement, 0.21–0.40 fair agreement, 0.41–0.60 moderate agreement, 0.61–0.80 substantial agreement, and 0.81-1.00 almost perfect agreement. Statistical significance of Kappa coefficients was tested using asymptotic standard errors under the null hypothesis (κ = 0), with p-values noted within each cell. Darker colors represent higher Kappa values, indicating better agreement. The inclusion of PTs-HDM improved inter-rater agreement across most groups, especially between more experienced participants. FAs, fibroadenomas; PTs, phyllodes tumors; -B, Benign; -M, Borderline/Malignant; PTs-HDM, phyllodes tumors hierarchical diagnosis model

PTs-HDM significantly improved diagnostic consistency among radiologists by standardizing lesion interpretation through probabilistic outputs and heatmap-based visual aids, fostering more uniform decision-making. As shown in Fig. 5, inter-radiologist agreement in binary classification of PTs vs. FAs, Kappa values improved from − 0.11 to 0.60 to 0.13–0.77, while ICC increased from 0.23 to 0.52. Similarly, for the hierarchical diagnosis (three-class classification) was initially poor, with Kappa values ranging from − 0.05 to 0.41 and an ICC of 0.19. After PTs-HDM integration, Kappa values increased to 0.12–0.65, and ICC rose to 0.45, indicating notable improvement. These findings demonstrate PTs-HDM’s ability to reduce diagnostic variability and enhance reliability by providing consistent, interpretable guidance that mitigates subjective differences.

### Subgroup analysis based on tumor size

To evaluate the influence of tumor size, patients were grouped into three categories: <2 cm (*n* = 77, 22 PTs), 2–4 cm (*n* = 64, 38 PTs), and ≥ 4 cm (*n* = 32, 31 PTs). Across all subgroups, PTs-HDM demonstrated high diagnostic accuracy (90.9%, 81.2%, and 90.6%, respectively). Sensitivity was robust (< 2 cm: 100.0%, 2–4 cm: 89.3%, ≥ 4 cm: 93.5%), while specificity showed greater variability (< 2 cm: 90.1%, 2–4 cm: 75.0%, ≥ 4 cm: 0.0%).

**Fig. 6:**
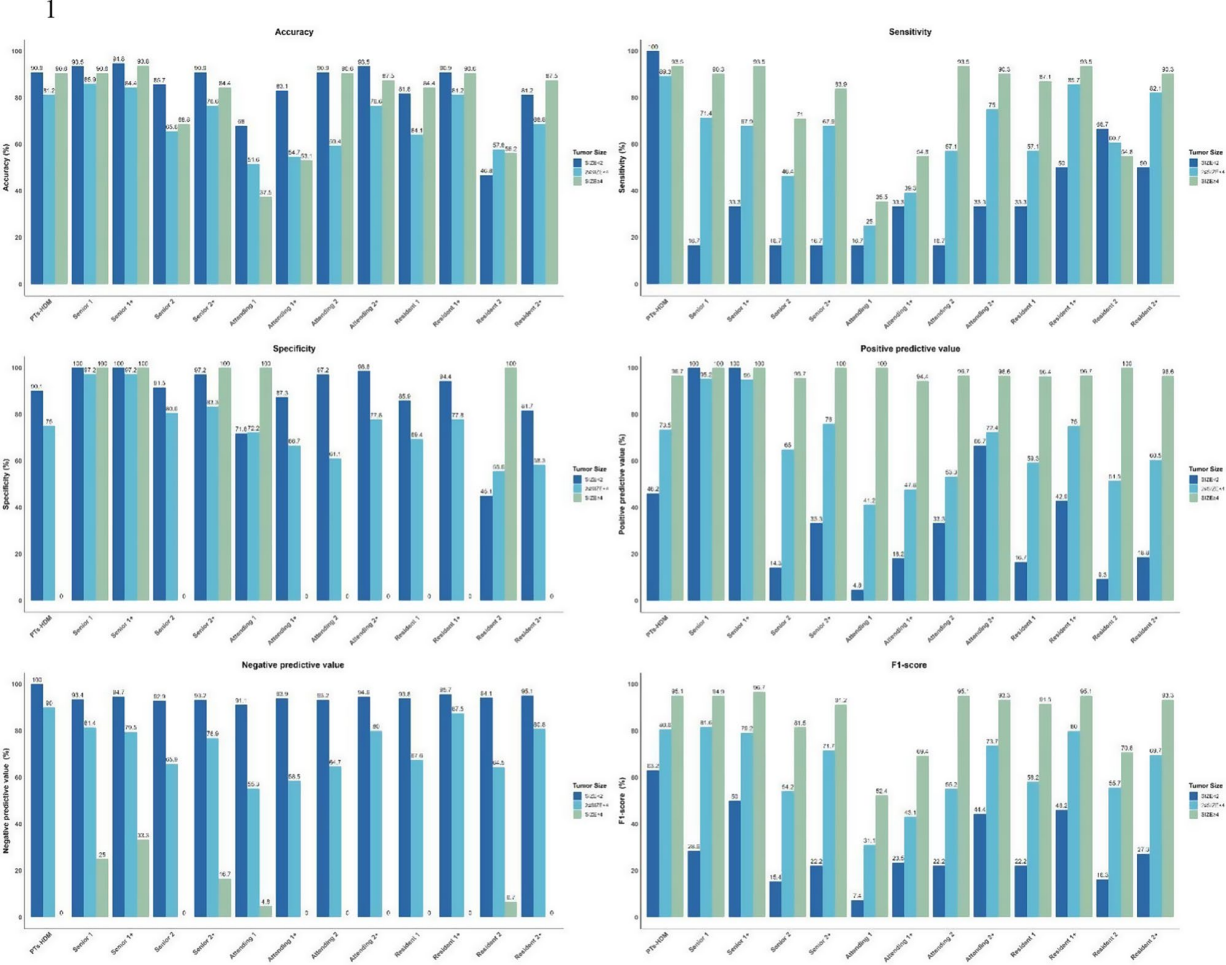
Bar charts illustrating the diagnostic performance of PTs-HDM and six radiologists for breast tumors of different sizes (< 2 cm, 2–4 cm, ≥ 4 cm), across six key metrics: Accuracy, Sensitivity, Specificity, Positive Predictive Value, Negative Predictive Value, and F1-score. PTs-HDM, phyllodes tumors hierarchical diagnosis model; FAs, fibroadenomas; PTs, phyllodes tumors; -B, Benign; -M, Borderline/Malignant; +, with PTs-HDM assistance

Radiologists’ performance also improved consistently with PTs-HDM integration. For tumors < 2 cm, accuracy increased from 46.8%-93.5–81.2-94.8%; for 2–4 cm lesions, from 51.6%-85.9–54.7-84.4%; and for ≥ 4 cm lesions, from 37.5%-90.6–53.1-93.8%. Notably, the most substantial F1-score improvements were observed in the < 2 cm subgroup, highlighting PTs-HDM’s potential in addressing diagnostic challenges for smaller lesions (Fig. 6). Comprehensive subgroup confusion matrices are available in the supplementary materials (Fig. S5-8), further supporting PTs-HDM’s role in enhancing diagnostic consistency and accuracy across tumor sizes.

## Discussion

PTs exhibit distinct biological behaviors from FAs, making accurate preoperative differentiation crucial for selecting appropriate surgical strategies. However, current diagnostic tools, such as imaging and biopsy, have significant limitations [18, 19]. This study presents the first comprehensive evaluation of PTs-HDM, a deep learning model for PT diagnosis and grading, using multicenter ultrasound data from 712 patients across 5 hospitals. The study also demonstrates PTs-HDM’s value in improving diagnostic accuracy and reducing discrepancies among radiologists of varying experience levels.

In the context of binary classification, Shi et al. [9]developed a DL model based on single-center ultrasound data to differentiate between PTs and FAs, achieving an AUC of 0.91. For PT grading, Basara et al. [27] analyzed texture features extracted from ultrasound images of 63 PT patients (41 benign, 12 borderline, and 10 malignant) to distinguish between benign PTs and borderline/malignant PTs. The AUC values for all independent factors discriminating between benign and malignant groups ranged from 0.65 to 0.75. Unlike prior single-center studies, our study incorporates a multicenter design and diverse ultrasound equipment, offering a more generalizable assessment of the model’s performance in real-world clinical settings. Multi-vendor ultrasound systems inherently induce feature-level heterogeneity in disease characterization due to device-specific variations in acoustic parameters (e.g., dynamic signal processing algorithms) [20, 21]. Despite these challenges, PTs-HDM demonstrated strong performance in both binary and three-class classification tasks for PT diagnosis, with AUC, accuracy, and sensitivity exceeding 0.8 in all evaluations.

In clinical practice, significant variability exists among radiologists in the preoperative diagnosis of PTs, which was further confirmed by our study. Our results demonstrated that the AUC values across six radiologists ranged from 0.507 to 0.872 (mean: 0.655) for binary classification and from 0.566 to 0.827 (mean: 0.712) for hierarchical diagnosis. Our study highlights the potential value of PTs-HDM as a preoperative diagnostic assistance tool in improving both the accuracy and consistency of diagnoses. With PTs-HDM assistance, the AUC values of the six radiologists improved to 0.629–0.880 (mean: 0.804) for binary classification and 0.725–0.837 (mean: 0.797) for hierarchical diagnosis. Moreover, the ICC among the six radiologists increased from 0.453 to 0.523 for binary classification and from 0.191 to 0.237 for hierarchical diagnosis. The pairwise kappa values between radiologists showed similar improvements, indicating that PTs-HDM assistance effectively reduced diagnostic disparities among radiologists with varying levels of experience.

We further analyzed the performance of both PTs-HDM and radiologists stratified by tumor size. PTs-HDM demonstrated significant value in assisting radiologists, although its performance varied among size groups. For tumors smaller than 2 cm, PTs-HDM achieved high diagnostic accuracy (90.9%) and sensitivity (100.0%), valuable for early-stage diagnoses, though its low PPV (46.2%) suggests careful interpretation of positive results. For medium-sized tumors (2–4 cm), the model showed balanced performance (accuracy 81.3%, sensitivity 89.3%), with specificity (75.0%) remaining an area for improvement. For tumors larger than 4 cm, while achieving high sensitivity (93.5%) and PPV (96.7%), the specificity (0%) performance indicates room for optimization. Moreover, the impact of AI assistance varied with radiologists’ experience levels, showing value in standardizing diagnoses among less experienced practitioners. For small tumors, less experienced radiologists (e.g., Resident 2) showed substantial improvement in diagnostic accuracy (46.8–79.2%). This finding suggests PTs-HDM’s potential in standardizing diagnostic procedures and reducing inter-observer variability. However, the varying improvement across size ranges emphasizes the importance of considering tumor size in AI system development and implementation.

Our study has several limitations. First, in real-world settings, the number of FA patients substantially exceeds that of PT patients. To ensure effective model training and adequately capture distinct imaging characteristics across different pathologies, we included an equal number of FA samples to match the number of PT samples [22–25]. Second, radiologists were limited to interpreting static two-dimensional grayscale images, whereas clinical diagnosis typically involves patient history, symptoms, and dynamic imaging information. Third, some radiologists showed minimal improvement with model assistance, possibly due to their already high diagnostic accuracy or conflicts between AI suggestions and clinical judgment. This occasional performance degradation suggests the need for more interpretable model recommendations [26, 27] and continuous learning mechanisms that incorporate radiologist feedback [28], thereby minimizing decision inconsistencies and enhancing diagnostic performance.

## Conclusions

PTs-HDM demonstrates strong performance in assisting radiologists with hierarchical diagnosis, enhancing consistency and accuracy particularly among radiologists with varying experience levels. Size-specific analysis reveals opportunities for optimization, particularly in improving PPV for small tumors and specificity for large tumors. Future development should focus on enhancing model performance across all tumor sizes while incorporating dynamic imaging features and feedback mechanisms.

## Electronic supplementary material

Below is the link to the electronic supplementary material.

Supplementary Material 1

## Data Availability

No datasets were generated or analysed during the current study.

## Abbreviations

AUC: The area under the receiver operating characteristic curve
CI: Confidence intervals
DL: Deep learning
FAs: Fibroadenomas
Grad CAM: Gradient-weighted Class Activation Mapping
ICC: Intraclass correlation coefficient
MRI: Magnetic resonance imaging
NPV: Negative predictive value
PPV: Positive predictive value
PTs: Phyllodes tumors
PTs-HDM: Phyllodes Tumors Hierarchical Diagnosis Model
ROC: Receiver operating characteristic
