# Supplementary material for: Hierarchical diagnosis of breast phyllodes tumors enabled by deep learning of ultrasound images: a retrospective multi-center study
Source: Cancer Imaging. 2025 May 8;25:61. doi: 10.1186/s40644-025-00879-9 (PMC12063467; doi:10.1186/s40644-025-00879-9)
Supplement: Supplementary file 1 — Supplementary Material 1 [file 40644_2025_879_MOESM1_ESM.docx]

**Supplementary material**

**Appendix S1: Participating organizations and related ethics approval numbers.**

**Appendix S2: Inclusion and exclusion criteria.**

**Appendix S3: Model training details.**

**Fig. S1 Twelve different ultrasound diagnostic instruments.**

**Fig. S2 DCA-supported results of PTs-HDM in therapeutic decision-making for hierarchical diagnosis of phyllodes tumors. a)** Differentiation between phyllodes tumors and fibroadenomas; **b)** Differentiation between borderline/malignant phyllodes tumors and benign phyllodes tumors. *DCA, Decision Curve Analysis; PTs-HDM, phyllodes tumors hierarchical diagnosis model.*

**Fig. S3 Visualization of the PTs-HDM using the Grad-CAM.** The regions of heightened response (highlighted in red) are also the regions that received greater attention from PTs-HDM This indicates that these regions hold more significance for predicting responses. *PTs-HDM, phyllodes tumors hierarchical diagnosis model; US, ultrasound; Pre-F, predicted fibroadenoma; true-F, true for fibroadenomas; Pre-B, predicted benign PTs; true-B, true for benign PTs; Pre-M, predicted borderline/malignant PTs, true-M, true for borderline/malignant PTs.*

**Fig. S4 Comparison of radiologists’ diagnostic performance radar charts under the PTs vs. FAs classification task.** *FAs, fibroadenomas; PTs, phyllodes tumors.*

**Fig. S5 Confusion matrix of PTs-HDM for binary classification of tumors of different sizes.** *PTs-HDM, phyllodes tumors hierarchical diagnosis model.*

**Fig. S6 Confusion matrix for binary classification of tumors less than 2 cm in size by six radiologists with and without PTs-HDM assistance.** *PTs-HDM, phyllodes tumors hierarchical diagnosis model.*

**Fig. S7 Confusion matrix for binary classification of tumors between 2 cm and 4 cm in size by six radiologists with and without PTs-HDM assistance.** *PTs-HDM, phyllodes tumors hierarchical diagnosis model.*

**Fig. S8 Confusion matrix for binary classification of tumors greater than or equal to 4 cm in size by six radiologists with and without PTs-HDM assistance.** *PTs-HDM, phyllodes tumors hierarchical diagnosis model.*

**Table S1 Diagnostic Performance of Sub-model for Diagnosis and Grading of Phyllodes Tumors/Diagnostic Performance of Sub-model.**

**Table S2. Results of the Delong test between the base models.**

**Table S3. Clinical and imaging characteristics of the training & validation set, and the external test set.**

**Table S4. Comparison of diagnostic performance between PTs-HDM and 6 radiologists.**

Appendix S1: Participating organizations and related ethics approval numbers

Hospital 1 (Zhejiang Cancer Hospital): IRB-2024-580

Hospital 2 (Ruijin Hospital-Shanghai Jiaotong University School of Medicine): IRB-2023-145

Hospital 3 (Zhejiang Xiaoshan Hospital): IRB-K2021-077

Hospital 4 (Dongyang People's Hospital): IRB-2024-YX-097

Hospital 5 (Sir Run Run Shaw Hospital): IRB-2022-354

Appendix S2: Model training details

The dataset was split into 80% for training and 20% for internal validation. The models included fully connected and classification layers after the feature extraction layers, which used pre-trained weights from the PASCAL challenge for transfer learning. The fully connected layers (with neurons configured as 256, 512, 1024, 512, 256, and 2, respectively) facilitated feature fusion. Dropout layers (50%) were used to prevent overfitting. Fine-tuning was applied to accelerate convergence for binary classification tasks. The models were trained for 300 epochs with a batch size of 32. ReLU activation functions were used between layers to mitigate overfitting, and the Softmax activation function was applied in the classification layer to output class probabilities.

**
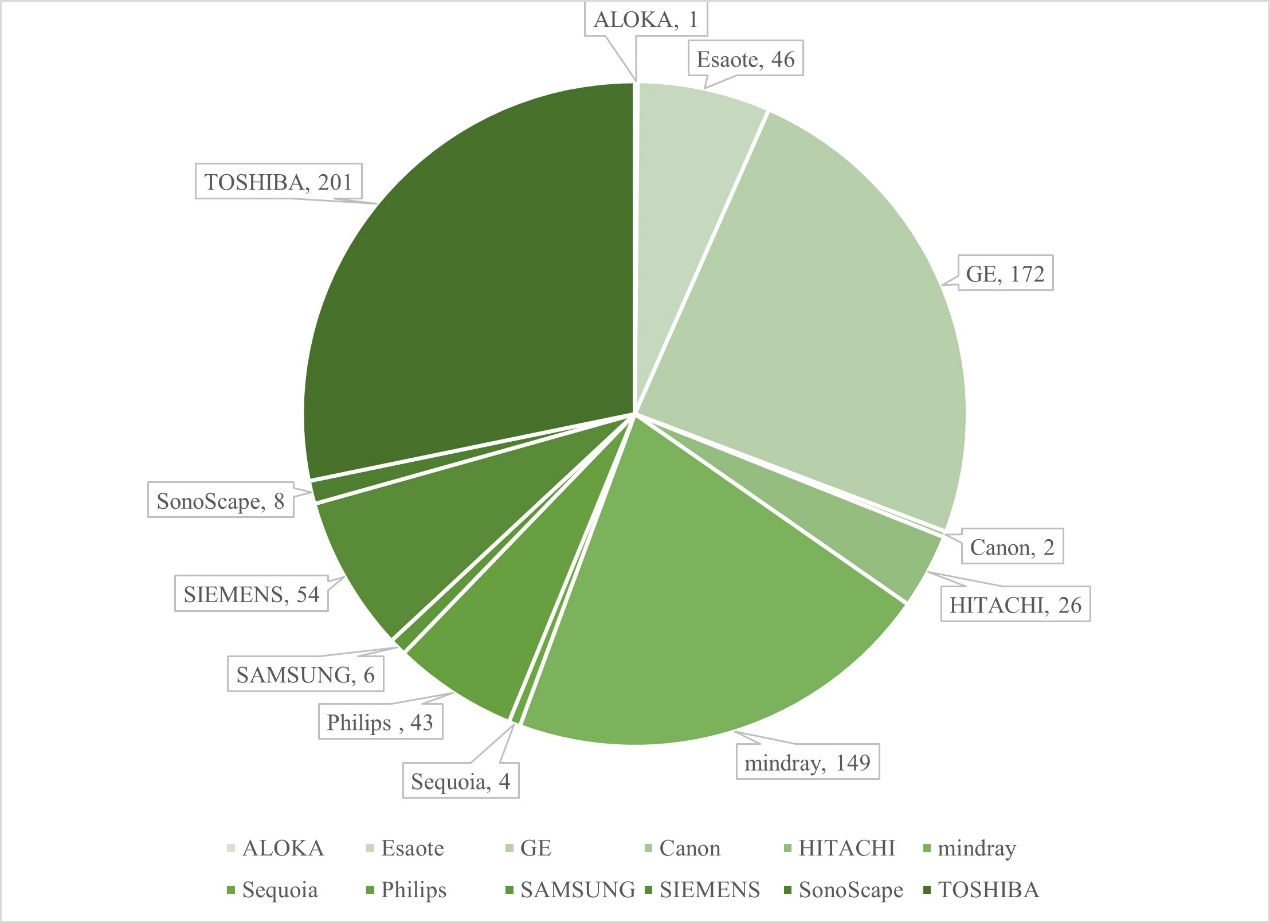
**

Fig. S1 Twelve different ultrasound diagnostic instruments.

**
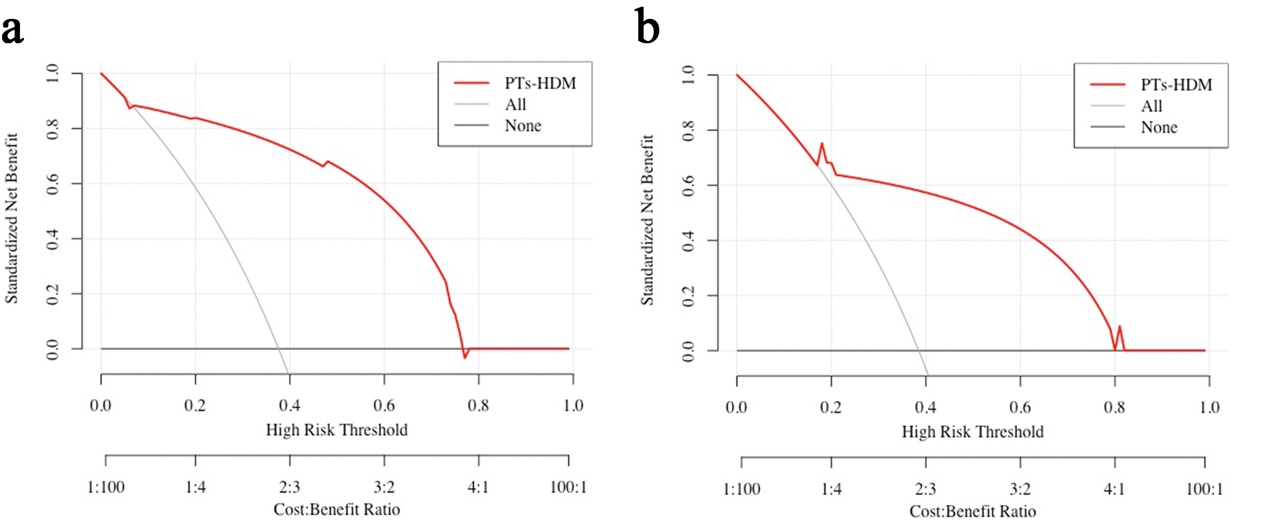
**

**Fig. S2 DCA-supported results of PTs-HDM in therapeutic decision-making for hierarchical diagnosis of phyllodes tumors. a)** Differentiation between phyllodes tumors and fibroadenomas; **b)** Differentiation between borderline/malignant phyllodes tumors and benign phyllodes tumors. *DCA, Decision Curve Analysis; PTs-HDM, phyllodes tumors hierarchical diagnosis model.*


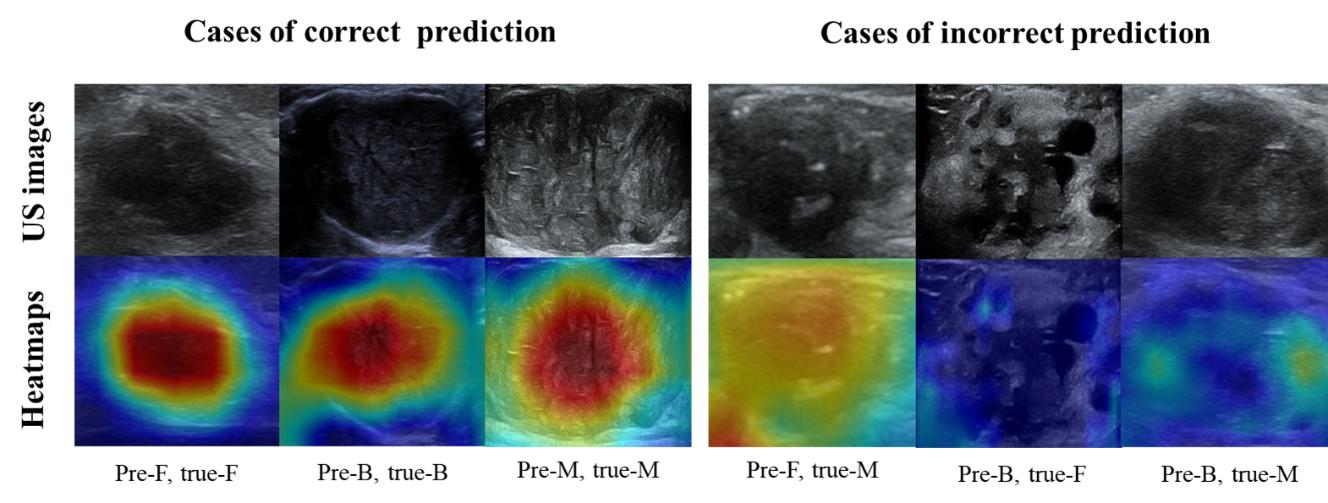
**Fig. S3: Visualization of the PTs-HDM using the Grad-CAM.** The regions of heightened response (highlighted in red) are also the regions that received greater attention from PTs-HDM This indicates that these regions hold more significance for predicting responses. *PTs-HDM, phyllodes tumors hierarchical diagnosis model; US, ultrasound; Pre-F, predicted fibroadenoma; true-F, true for fibroadenomas; Pre-B, predicted benign PTs; true-B, true for benign PTs; Pre-M, predicted borderline/malignant PTs, true-M, true for borderline/malignant PTs.*

*
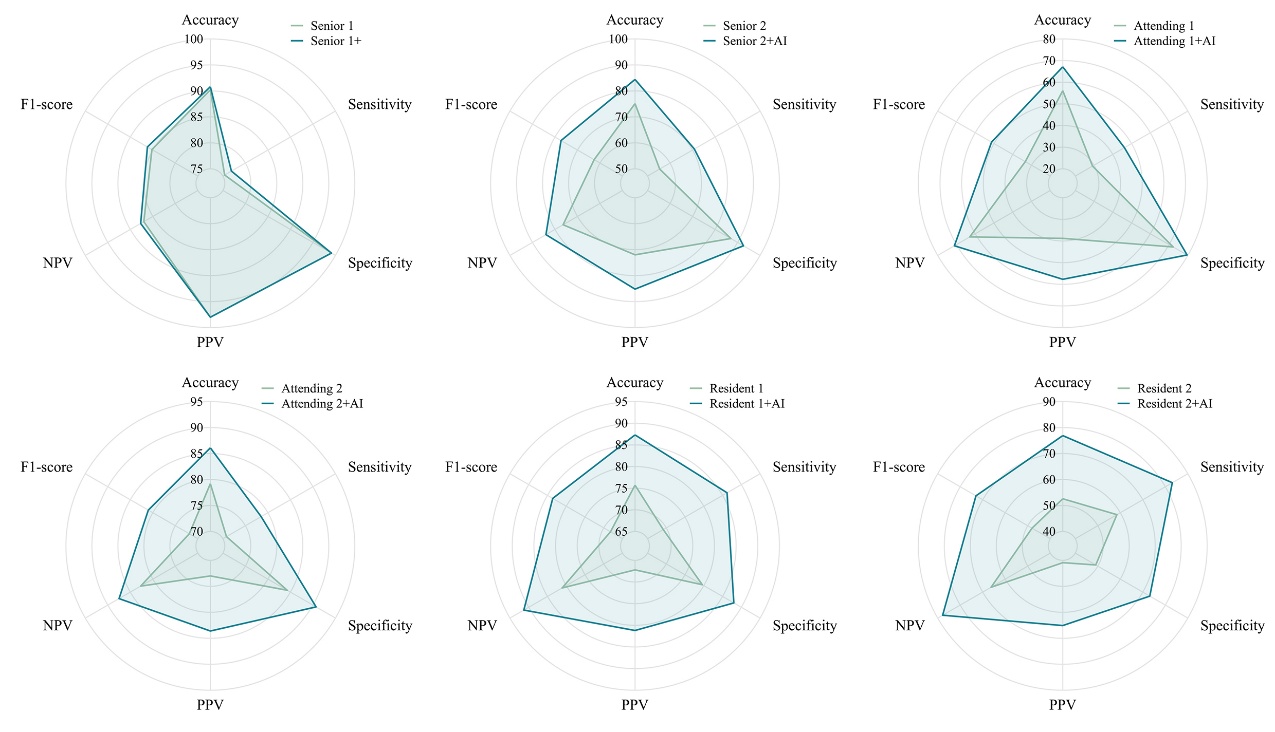
*

#### **Fig. S4: Comparison of radiologists’ diagnostic performance radar charts under the PTs vs. FAs classification task.** *+ indicates with PTs-HDM assistance; PTs-HDM, phyllodes tumors hierarchical diagnosis model.*


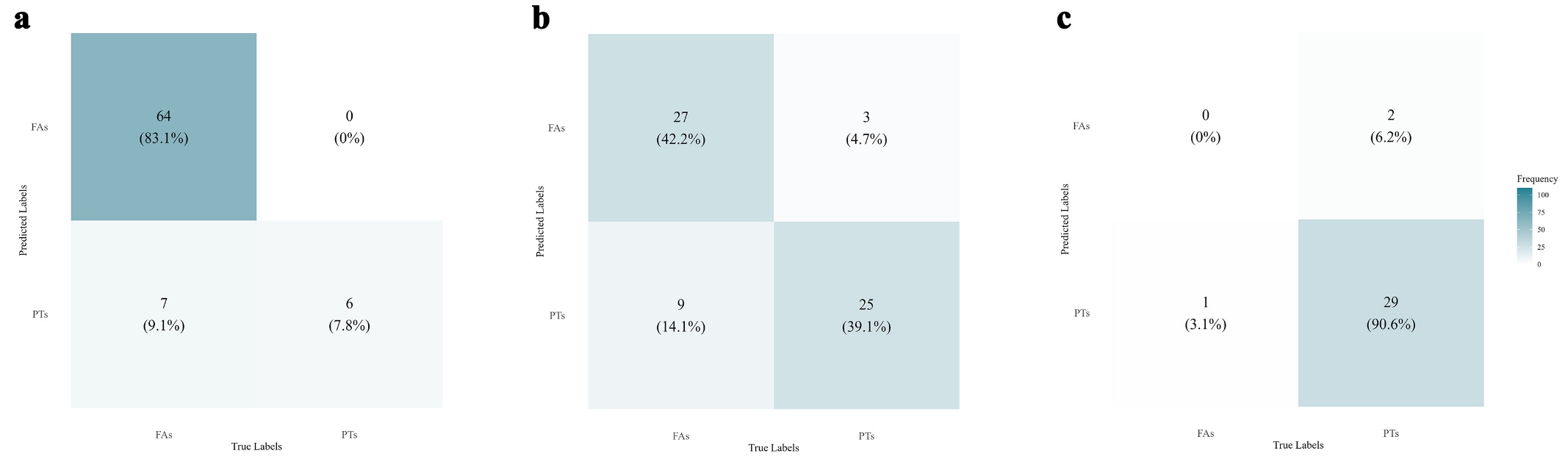


Fig. S5 Confusion matrix of PTs-HDM for binary classification of tumors of different sizes. a) Tumor less than 2cm; b) Tumor between 2cm-4cm; c) Tumor greater than or equal to 4cm. *PTs-HDM, phyllodes tumors hierarchical diagnosis model.*


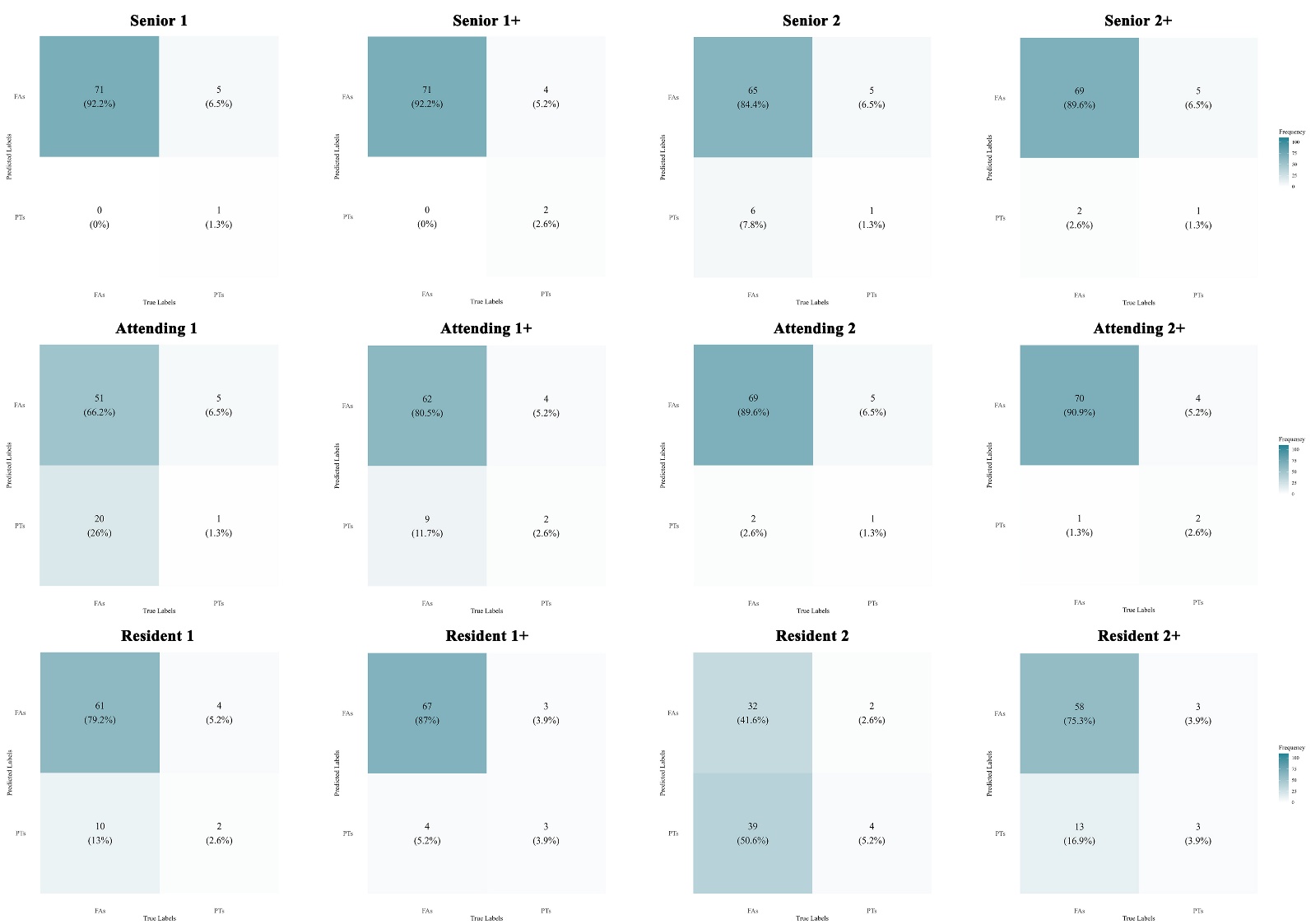


Fig. S6 Confusion matrix for binary classification of tumors less than 2 cm in size by six radiologists with and without PTs-HDM assistance. *PTs-HDM, phyllodes tumors hierarchical diagnosis model.*


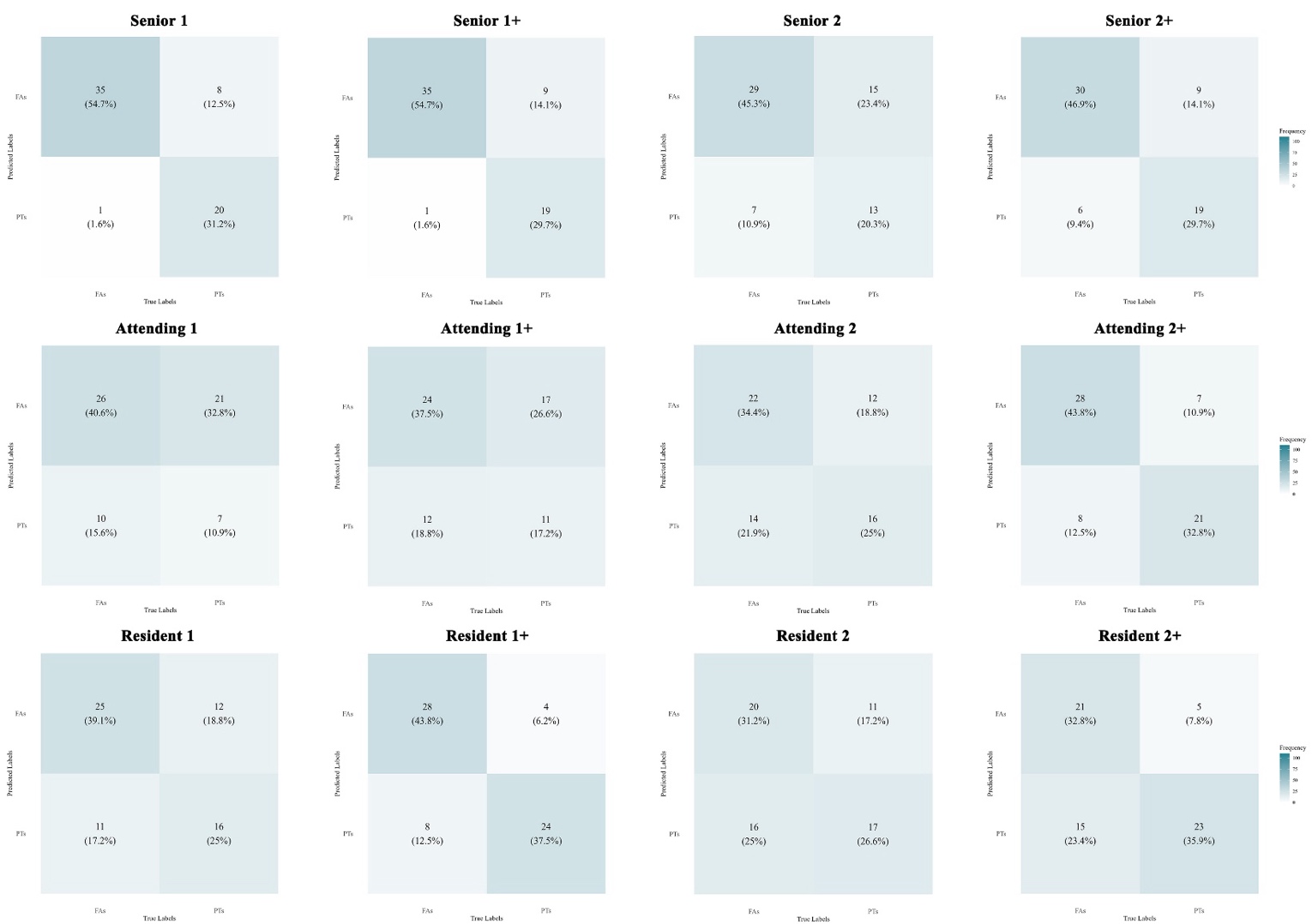
Fig. S7 Confusion matrix for binary classification of tumors between 2 cm and 4 cm in size by six radiologists with and without PTs-HDM assistance. *PTs-HDM, phyllodes tumors hierarchical diagnosis model.*


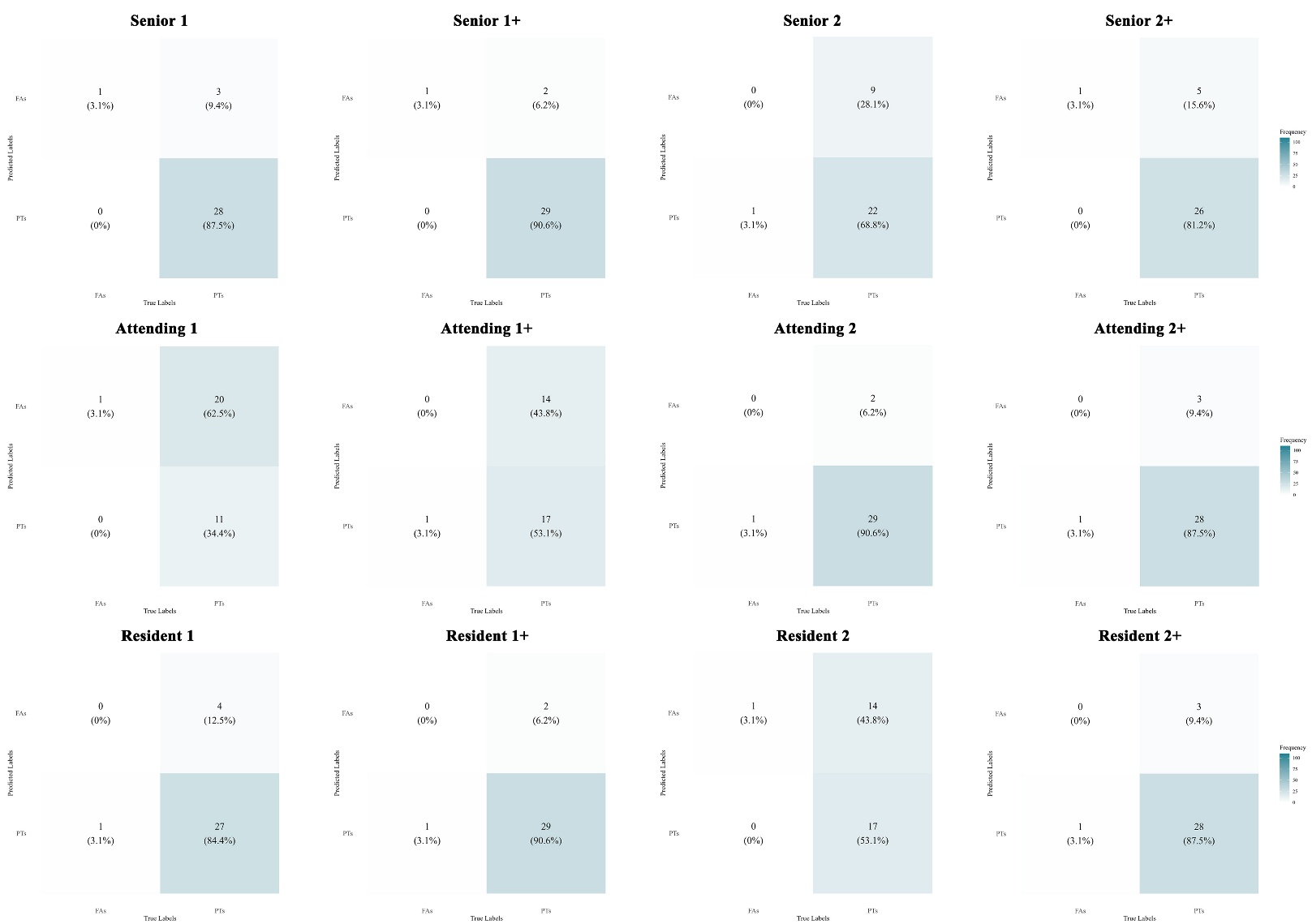


F**ig. S8 Confusion matrix for binary classification of tumors greater than or equal to 4 cm in size by six radiologists with and without PTs-HDM assistance.** *PTs-HDM, phyllodes tumors hierarchical diagnosis model.*

| Table S1 Diagnostic Performance of Sub-model for Diagnosis and Grading of Phyllodes Tumors/Diagnostic Performance of Sub-model | | | | | | | | |
| --- | --- | --- | --- | --- | --- | --- | --- | --- |
|  | **AUC** | **Accuracy** | **Sensitivity** | **Specificity** | **PPV** | **NPV** | **F1-score** |  |
| **PTs diagnosis** |  |  |  |  |  |  |  |  |
| DenseNet121 | 0.857 (0.827, 0.887) | 86.7 (81.6, 91.8) | 87.0 (82.0, 92.0) | 86.2 (81.0, 91.3) | 91.3 (87.1, 95.5) | 80.0 (74.0, 86.0) | 83.0 (77.4, 88.6) |  |
| InceptionV3 | 0.832 (0.801, 0.864) | 64.7 (57.6, 71.9) | 43.5 (36.1, 50.9) | 100.0 (100.0, 100.0) | 100.0 (100.0, 100.0) | 51.6 (44.1, 59.0) | 68.1 (61.1, 75.0) |  |
| MobileNetV22 | 0.880 (0.853, 0.908) | 80.3 (74.4, 86.3) | 74.1 (67.5, 80.6) | 90.8 (86.5, 95.1) | 93.0 (89.2, 96.8) | 67.8 (60.9, 74.8) | 77.6 (71.4, 83.8) |  |
| ResNet50V2 | 0.852 (0.822, 0.882) | 85.5 (80.3, 90.8) | 93.5 (89.8, 97.2) | 72.3 (65.6, 79.0) | 84.9 (79.5, 90.2) | 87.0 (82.0, 92.0) | 79.0 (72.9, 85.1) |  |
| **Xception** | **0.893 (0.867, 0.919)** | **86.1 (81.0, 91.3)** | **83.3 (77.8, 88.9)** | **90.8 (86.5, 95.1)** | **93.8 (90.1, 97.4)** | **76.6 (70.3, 82.9)** | **83.0 (77.5, 88.7)** |  |
| **PTs grading** |  |  |  |  |  |  |  |  |
| DenseNet121 | 0.707 (0.646, 0.768) | 72.3 (61.4, 83.2) | 60.5 (48.6, 72.4) | 88.9 (81.2, 96.5) | 88.5 (80.7, 96.2) | 61.5 (49.7, 73.4) | 72.7 (61.9, 83.6) |  |
| InceptionV3 | 0.770 (0.714, 0.826) | 80.0 (70.3, 89.7) | 81.6 (72.2, 91.0) | 77.8 (67.7, 87.9) | 83.8 (74.8, 92.7) | 75.0 (64.5, 85.5) | 76.4 (66.0, 86.7) |  |
| MobileNetV22 | 0.734 (0.675, 0.793) | 73.8 (63.2, 84.5) | 57.9 (45.9, 69.9) | 96.3 (91.7, 100.9) | 95.7 (90.7, 100.6) | 61.9 (50.1, 73.7) | 75.4 (64.9, 85.8) |  |
| **ResNet50V2** | **0.869 (0.824, 0.914)** | **80.0 (70.3, 89.7)** | **86.8 (78.6, 95.1)** | **70.4 (59.3, 81.5)** | **80.5 (70.9, 90.1)** | **79.2 (69.3, 89.0)** | **74.5 (63.9, 85.1)** |  |
| Xception | 0.847 (0.799, 0.895) | 81.5 (72.1, 91.0) | 71.1 (60.0, 82.1) | 96.3 (91.7, 100.9) | 96.4 (91.9, 100.9) | 70.3 (59.2, 81.4) | 81.2 (71.8, 90.7) |  |
| *The data in brackets represent the 95% confidence intervals. AUC****,*** *the area under the receiver operating characteristic curve; PPV, positive predictive value; NPV, negative predictive value* | | | | | | | |  |

| Table S2. Results of the Delong test between the base models | | | | | |
| --- | --- | --- | --- | --- | --- |
|  | **DenseNet121** | **InceptionV3** | **MobileNetV22** | **ResNet50V2** | **Xception** |
| **PTs diagnosis** |  |  |  |  |  |
| DenseNet121 | 1.00 | < 0.001 | 0.17 | 0.28 | 0.86 |
| InceptionV3 | < 0.001 | 1.00 | < 0.001 | < 0.001 | < 0.001 |
| MobileNetV22 | 0.17 | < 0.001 | 1.00 | 0.88 | 0.05 |
| ResNet50V2 | 0.28 | < 0.001 | 0.88 | 1.00 | 0.20 |
| Xception | 0.86 | < 0.001 | 0.05 | 0.20 | 1.00 |
| **PTs grading** |  |  |  |  |  |
| DenseNet121 | 1.00 | 0.46 | 0.72 | 0.56 | 0.18 |
| InceptionV3 | 0.46 | 1.00 | 0.69 | 0.85 | 0.48 |
| MobileNetV22 | 0.72 | 0.69 | 1.00 | 0.81 | 0.22 |
| ResNet50V2 | 0.56 | 0.85 | 0.81 | 1.00 | 0.43 |
| Xception | 0.18 | 0.48 | 0.22 | 0.43 | 1.00 |
| *Using the Delong test to compare the AUCs of different models, if the p-value is less than 0.05, it indicates that there is a statistically significant difference in their predictive abilities.* | | | | | |

| Table S3. Clinical and imaging characteristics of the training & validation set, and the external test set. | | | | | | | | | | | | |  |
| --- | --- | --- | --- | --- | --- | --- | --- | --- | --- | --- | --- | --- | --- |
| **Characteristics** | **Training & Validation cohort** | | | | |  | | **Test cohort** | | | | |  |
|  | **Benign**  **PTs** | **Borderline PTs** | **Malignant PTs** | **PTs-all** | **FAs** |  | **Benign**  **PTs** | | **Borderline PTs** | **Malignant PTs** | **PTs-all** | **FAs** | |
| **Patients (n)** | 120 | 95 | 32 | 247 | 292 |  | 38 | | 15 | 12 | 65 | 108 | |
| **Images (n)** | 312 | 208 | 68 | 588 | 593 |  | 132 | | 48 | 43 | 223 | 314 | |
| **Age (y), M (Q₁, Q₃)** | 43.5  (36.0, 48.0) | 50.5  (45.0, 56.0) | 48.5  (39.25, 51.75) | 46.0  (39.0, 52.0) | 38.0  (27.0, 48.0) |  | 43.5  (38.3, 50.8) | | 53.0  (44.50, 57.0) | 49.0  (43.50, 58.0) | 46.0  (40.0, 54.0) | 39.0  (31.8, 48.0) | |
| **Menstrual status** **(n, %)** |  |  |  |  |  |  |  | |  |  |  |  | |
| Postmenopausal | 22 (18.3) | 40 (42.1) | 9 (28.1) | 71 (28.7) | 44 (15.1) |  | 10 (26.3) | | 8 (53.3) | 5 (41.7) | 23 (35.4) | 19 (17.6) | |
| Premenopausal | 98 (81.7) | 55 (57.9) | 23 (71.9) | 176 (71.3) | 248 (84.9) |  | 28 (73.7) | | 7 (46.7) | 7 (58.3) | 42 (64.6) | 89 (82.4) | |
| **Lesion diameter (mm), M (Q₁, Q₃)** | 29.5  (23.0,37.25) | 35.0  (26.0,47.0) | 33.5  (25.75, 43.0) | 32.0  (24.0, 41.0) | 19.0  (14.0, 26.0) |  | 34.50  (24.25, 47.0) | | 41.0  (31.50, 58.50) | 44.0  (31.75, 55.75) | 37.0  (29.0, 53.0) | 17.0  (12.0, 21.5) | |
| *PTs = phyllodes tumors; FAs = fibroadenoma;* *M: Median, Q₁: 1st Quartile, Q₃: 3st Quartile* | | | | | | | | | | | | |  |

| Table S4. Comparison of diagnostic performance between PTs-HDM and 6 radiologists | | | | | |
| --- | --- | --- | --- | --- | --- |
|  | **AUC-weighted** | **Accuracy-weighted** | **Recall-weighted** | **Precision-weighted** | **F1-weighted** |
| **PTs-HDM** | 0.858 (0.810, 0.906) | 81.0 (74.6, 86.7) | 81.0 (75.1, 86.7) | 84.5 (78.9, 89.4) | 82.0 (76.8, 86.8) |
| **Senior 1** | 0.788 (0.736, 0.842) | 76.9 (69.9, 82.7) | 76.9 (70.5, 82.7) | 66.4 (57.8, 75.3) | 71.1 (63.1, 78.5) |
| **Senior 1+** | 0.791 (0.742, 0.841) | 76.9 (71.1, 82.7) | 76.9 (69.9, 82.7) | 72.8 (60.2, 84.8) | 71.7 (64.3, 79.1) |
| **Senior 2** | 0.670 (0.615, 0.732) | 66.4 (59.0, 73.4) | 66.4 (59.0, 73.4) | 70.2 (53.3, 78.1) | 62.8 (55.3, 70.2) |
| **Senior 2+** | 0.763 (0.708, 0.819) | 74.6 (68.2, 80.9) | 74.7 (68.2, 80.9) | 73.7 (66.1, 80.5) | 73.2 (66.1, 80.2) |
| **Senior Mean** | 0.729 (0.676, 0.787) | 71.7 (64.5, 78.1) | 71.7 (64.8, 78.1) | 68.3 (55.6, 76.7) | 67.0 (59.2, 74.4) |
| **Senior Mean+** | 0.777 (0.725, 0.830) | 75.8 (69.7, 81.8) | 75.8 (69.1, 81.8) | 73.3 (63.2, 82.7) | 72.5 (65.2, 79.7) |
| **Attending 1** | 0.512 (0.454, 0.571) | 51.6 (44.5, 59.0) | 51.5 (43.9, 59.0) | 48.0 (38.6, 56.8) | 48.9 (40.7, 57.0) |
| **Attending 1+** | 0.626 (0.560, 0.689) | 62.8 (55.5, 69.9) | 62.7 (55.5, 69.9) | 60.7 (52.4, 68.7) | 61.3 (53.0, 69.1) |
| **Attending 2** | 0.738 (0.679, 0.795) | 70.6 (64.1, 76.9) | 70.5 (63.6, 77.5) | 70.5 (63.2, 77.5) | 70.4 (62.7, 78.1) |
| **Attending 2+** | 0.783 (0.730, 0.839) | 74.6 (67.6, 80.9) | 74.5 (68.2, 80.3) | 74.0 (66.6, 80.4) | 74.1 (66.9, 80.7) |
| **Attending Mean** | 0.625 (0.567, 0.683) | 61.1 (54.3, 68.0) | 61.0 (53.8, 68.3) | 59.3 (50.9, 67.2) | 59.7 (51.7, 67.6) |
| **Attending Mean+** | 0.705 (0.645, 0.764) | 68.7 (61.6, 75.4) | 68.6 (61.9, 75.1) | 67.4 (59.5, 74.6) | 67.7 (60.0, 74.9) |
| **Resident 1** | 0.678 (0.619, 0.739) | 62.0 (54.9, 68.8) | 61.9 (54.3, 69.4) | 63.2 (55.4, 71.1) | 62.1 (54.5, 69.9) |
| **Resident 1+** | 0.825 (0.771, 0.879) | 78.1 (71.7, 84.4) | 78.3 (72.3, 84.4) | 80.2 (74.0, 86.0) | 78.5 (72.2, 84.3) |
| **Resident 2** | 0.539 (0.478, 0.595) | 42.4 (35.3, 49.7) | 42.2 (34.7, 50.3) | 50.5 (40.7, 60.2) | 43.4 (36.1, 50.8) |
| **Resident 2+** | 0.764 (0.700, 0.827) | 69.1 (61.8, 75.7) | 69.4 (61.8, 76.3) | 74.1 (67.4, 80.6) | 70.7 (64.6, 77.0) |
| **Resident Mean** | 0.609 (0.549, 0.667) | 52.2 (45.1, 59.3) | 52.1 (44.5, 59.9) | 56.9 (48.1, 65.7) | 52.8 (45.3, 60.4) |
| **Resident Mean+** | 0.795 (0.736, 0.853) | 73.6 (66.8, 80.1) | 73.9 (67.1, 80.4) | 77.2 (70.7, 83.3) | 74.6 (68.4, 80.7) |
| *The data in brackets represent the 95% confidence intervals. ACC = accuracy; + AI indicates with PTs-HDM assistance. The upward arrow (↑) represents indicators that improved owing to AI assistance; PTs-HDM, phyllodes tumors hierarchical diagnosis model.* | | | | | |
